# Cortical growth from infancy to adolescence in preterm and term-born children

**DOI:** 10.1093/brain/awad348

**Published:** 2023-10-10

**Authors:** Claire E Kelly, Deanne K Thompson, Chris L Adamson, Gareth Ball, Thijs Dhollander, Richard Beare, Lillian G Matthews, Bonnie Alexander, Jeanie L Y Cheong, Lex W Doyle, Peter J Anderson, Terrie E Inder

**Affiliations:** Turner Institute for Brain and Mental Health, School of Psychological Sciences, Monash University, Melbourne, VIC 3800, Australia; Victorian Infant Brain Studies (VIBeS), Murdoch Children’s Research Institute, Melbourne, VIC 3052, Australia; Developmental Imaging, Murdoch Children’s Research Institute, Melbourne, VIC 3052, Australia; Turner Institute for Brain and Mental Health, School of Psychological Sciences, Monash University, Melbourne, VIC 3800, Australia; Victorian Infant Brain Studies (VIBeS), Murdoch Children’s Research Institute, Melbourne, VIC 3052, Australia; Developmental Imaging, Murdoch Children’s Research Institute, Melbourne, VIC 3052, Australia; Department of Paediatrics, The University of Melbourne, Melbourne, VIC 3052, Australia; Developmental Imaging, Murdoch Children’s Research Institute, Melbourne, VIC 3052, Australia; Developmental Imaging, Murdoch Children’s Research Institute, Melbourne, VIC 3052, Australia; Department of Paediatrics, The University of Melbourne, Melbourne, VIC 3052, Australia; Developmental Imaging, Murdoch Children’s Research Institute, Melbourne, VIC 3052, Australia; Developmental Imaging, Murdoch Children’s Research Institute, Melbourne, VIC 3052, Australia; National Centre for Healthy Ageing and Peninsula Clinical School, Faculty of Medicine, Monash University, Melbourne, VIC 3199, Australia; Turner Institute for Brain and Mental Health, School of Psychological Sciences, Monash University, Melbourne, VIC 3800, Australia; Victorian Infant Brain Studies (VIBeS), Murdoch Children’s Research Institute, Melbourne, VIC 3052, Australia; Department of Pediatric Newborn Medicine, Brigham and Women’s Hospital, Harvard Medical School, Boston, MA 02115, USA; Developmental Imaging, Murdoch Children’s Research Institute, Melbourne, VIC 3052, Australia; Department of Neurosurgery, The Royal Children’s Hospital, Melbourne, VIC 3052, Australia; Victorian Infant Brain Studies (VIBeS), Murdoch Children’s Research Institute, Melbourne, VIC 3052, Australia; Department of Paediatrics, The University of Melbourne, Melbourne, VIC 3052, Australia; Newborn Research, The Royal Women’s Hospital, Melbourne, VIC 3052, Australia; Department of Obstetrics and Gynaecology, The University of Melbourne, Melbourne, VIC 3052, Australia; Victorian Infant Brain Studies (VIBeS), Murdoch Children’s Research Institute, Melbourne, VIC 3052, Australia; Newborn Research, The Royal Women’s Hospital, Melbourne, VIC 3052, Australia; Department of Obstetrics and Gynaecology, The University of Melbourne, Melbourne, VIC 3052, Australia; Turner Institute for Brain and Mental Health, School of Psychological Sciences, Monash University, Melbourne, VIC 3800, Australia; Victorian Infant Brain Studies (VIBeS), Murdoch Children’s Research Institute, Melbourne, VIC 3052, Australia; Center for Neonatal Research, Children's Hospital of Orange County, Orange, CA 92868, USA; Department of Pediatrics, University of California, Irvine, Irvine, CA 92697, USA

**Keywords:** very preterm birth, neurodevelopment, longitudinal, magnetic resonance imaging

## Abstract

Early life experiences can exert a significant influence on cortical and cognitive development. Very preterm birth exposes infants to several adverse environmental factors during hospital admission, which affect cortical architecture. However, the subsequent consequence of very preterm birth on cortical growth from infancy to adolescence has never been defined; despite knowledge of critical periods during childhood for establishment of cortical networks. Our aims were to: chart typical longitudinal cortical development and sex differences in cortical development from birth to adolescence in healthy term-born children; estimate differences in cortical development between children born at term and very preterm; and estimate differences in cortical development between children with normal and impaired cognition in adolescence.

This longitudinal cohort study included children born at term (≥37 weeks’ gestation) and very preterm (<30 weeks’ gestation) with MRI scans at ages 0, 7 and 13 years (*n* = 66 term-born participants comprising 34 with one scan, 18 with two scans and 14 with three scans; *n* = 201 very preterm participants comprising 56 with one scan, 88 with two scans and 57 with three scans). Cognitive assessments were performed at age 13 years. Cortical surface reconstruction and parcellation were performed with state-of-the-art, equivalent MRI analysis pipelines for all time points, resulting in longitudinal cortical volume, surface area and thickness measurements for 62 cortical regions. Developmental trajectories for each region were modelled in term-born children, contrasted between children born at term and very preterm, and contrasted between all children with normal and impaired cognition.

In typically developing term-born children, we documented anticipated patterns of rapidly increasing cortical volume, area and thickness in early childhood, followed by more subtle changes in later childhood, with smaller cortical size in females than males. In contrast, children born very preterm exhibited increasingly reduced cortical volumes, relative to term-born children, particularly during ages 0–7 years in temporal cortical regions. This reduction in cortical volume in children born very preterm was largely driven by increasingly reduced cortical thickness rather than area. This resulted in amplified cortical volume and thickness reductions by age 13 years in individuals born very preterm. Alterations in cortical thickness development were found in children with impaired language and memory.

This study shows that the neurobiological impact of very preterm birth on cortical growth is amplified from infancy to adolescence. These data further inform the long-lasting impact on cortical development from very preterm birth, providing broader insights into neurodevelopmental consequences of early life experiences.

## Teaser

Early life experiences can affect brain development. Infants born very preterm are exposed to many stressors early in life, yet much remains unclear around the effects of preterm birth on cortical development. Kelly *et al.* show that the impact of very preterm birth on cortical growth is amplified from infancy to adolescence.

## Introduction

The cerebral cortex is a highly convoluted sheet of neural tissue, which can be studied *in vivo* by using structural MRI to obtain high-resolution maps of cortical anatomy, including cortical volume, surface area and thickness.^1^ Cortical surface area expansion occurs with the rapid tangential growth of the outer layer of the cortical surface, a process associated with the differentiation of the neurons of the cortical plate, the growth of their cell bodies and of the surrounding neuropil (formation of axons, dendrites and synapses),^2^ whereas cortical thickening may be more primarily driven by neuronal production within cortical columns.^3^ Patterns of cortical folding may result from the complex dynamics of mechanical expansion of the outer layer of the cortex overlayed on a ‘protomap’ of folding patterns established from the subventricular zone, related to migration patterns of neurons into the cortex leading to fundamental sulcal roots and pits.^4^ Longitudinal MRI studies have shown that cortical area and thickness undergo rapid changes over the first two postnatal years, followed by more protracted changes throughout childhood and adolescence.^5,6^ There are known sex differences in cortical structure during childhood, with males having larger cortical size and surface area than females.^5,6^

Early life stress or adversity can affect both the process and outcome of cortical development.^7,8^ Infants born very preterm (<32 weeks of gestation) are exposed to differing physical, psychological and sensorial stressors (such as medical procedures, excessive noise and light, and maternal separation) during their care in the newborn intensive care unit.^9^ Exposure to such adverse environmental factors during early sensitive periods may alter typical cortical developmental processes in infants born very preterm.

Cortical development occurs alongside rapid cognitive, affective and behavioural development. The trajectory of longitudinal cortical thickness development has been related to cognitive abilities in typically developing children,^10^ and deviations in longitudinal cortical thickness development have been observed in individuals with neurodevelopmental and psychiatric disorders.^10^ Children born preterm are at higher risk of experiencing cognitive impairments, with specific areas of particular concern being language, memory and learning impairments, which are known to constrain academic achievement, social interactions, and mental health.^11-13^

Despite these observations, there have been no longitudinal studies of cortical development encompassing the entire period from birth to adolescence in the same individuals; limiting our ability to understand influences of early experiences on cortical and cognitive development. Prior studies of cortical development used cross-sectional MRI designs^14^ or longitudinal MRI designs in specific age windows, with newborns and young infants (approximately aged 0–2 years)^15,16^ being investigated separately to older children, adolescents or young adults (approximately aged 4 years or older).^17^ This lack of longitudinal studies has been due to the absence of both a relevant longitudinal cohort and appropriate MRI processing technologies. The recent advent of cortical surface extraction tools and parcellation schemes specific to neonatal MRI^18-20^ has allowed for automatic measurement of neonatal cortical anatomy with high accuracy and equivalency to tools commonly used in older child and adult MRI scans.^21^ This, in turn, has enabled integration of longitudinal neonatal, childhood and adolescent regional cortical surface-based volume, area and thickness data.

A large body of literature has described cortical surface extraction and the cortical folding process in the newborn period in those born preterm and term, which correlates with behavioural outcomes.^22-26^ Cortical volume, area and thickness have also been investigated at cross-sectional time points in individuals born very preterm. These prior cross-sectional studies documented that infants born preterm exhibited lower cortical area in frontal, parietal and temporal regions,^27-29^ and higher cortical thickness in frontal, parietal and insula regions^30^ compared with term-born infants at hospital discharge (term-equivalent age). After hospital discharge, at cross-sectional time points in childhood, those born preterm exhibited lower cortical area across frontal, parietal and temporal regions,^31,32^ lower cortical thickness in temporal and parietal regions, and higher cortical thickness in frontal and occipital regions,^31,32^ compared with those born at term. Some studies have looked at longitudinal cortical volume changes in children born preterm,^33-37^ or longitudinal cortical area and thickness changes in children born preterm in specific age windows.^31,38,39^ Taken together, prior studies suggest there may be particular vulnerability following preterm birth of temporal regions, which are thought to have a role in language development and memory formation.^34,40,41^ However, due to the lack of longitudinal studies spanning infancy to adolescence, it remains unknown how cortical area and thickness alterations observed in infants born preterm progress longitudinally from term-equivalent age to adolescence and relate to language, memory and learning functioning. Prior studies were unable to distinguish whether cortical alterations remain stable, worsen, or improve from infancy to later in childhood following very preterm birth. That is, it remains unclear how, as infants develop, their early experiences become embedded in the anatomy of their cortex to shape their lifelong neurological health. This lack of understanding could lead us to underestimate the importance of the earliest stages of life and miss opportunities for interventions.

This study aimed to examine typical longitudinal cortical volume, area and thickness development from birth to adolescence, and estimate differences in cortical development between sexes, in healthy term-born children. Then, this study aimed to estimate differences in cortical development between children born at term and very preterm. It was hypothesized that children born very preterm would exhibit both altered cortical structure and altered longitudinal cortical developmental trajectories; meaning that cortical alterations would become further amplified through childhood in this population. Finally, this study aimed to estimate differences in longitudinal cortical development between all children with normal and impaired language, memory and learning functioning in adolescence. It was hypothesized that longitudinal cortical development would be altered in children who have impaired language, memory and learning function, irrespective of group (very preterm or term-born).

## Materials and methods

### Participants and procedures

This study was based on a longitudinal cohort of children born at term and very preterm. Participants were recruited as part of the Victorian Infant Brain Study (VIBeS), a prospective longitudinal cohort study of infants born preterm (specifically, very preterm at <30 weeks of gestation or <1250 g birth weight) and at term (≥37 weeks of gestation) between 2001 and 2004 in Melbourne, Australia. A total of *n* = 224 infants born very preterm without congenital abnormalities were recruited from the Royal Women’s Hospital, Melbourne. A total of *n* = 76 infants born at term were recruited from postnatal wards and via response to advertisements in the neonatal period, or from Maternal and Child Health Centres at age 2 years. Perinatal medical data including key, clinically important variables of gestational age at birth, birth weight, sex, major neonatal brain injuries and bronchopulmonary dysplasia were collected by chart review. Infants underwent MRI scans without sedation at the Royal Children’s Hospital, Melbourne, at term-equivalent age (age 0 years) and returned for follow-up MRI scans without sedation at the same site at 7 years of age (occurring between April 2008 and August 2011) and 13 years of age (occurring between October 2014 and January 2017). Neuropsychological assessments were conducted at the Royal Childrens Hospital, Melbourne, at 13 years of age. The study was approved by the Human Research Ethics Committees of the participating hospitals (The Royal Women’s Hospital and The Royal Children’s Hospital, Melbourne). Parental written informed consent was obtained for all participants. The Supplementary material provides a detailed description of participant numbers at each follow-up, including a participant flow chart (Supplementary Fig. 1 and Supplementary Table 1).

### Brain MRI analysis

Of the MRI sequences acquired at each follow-up, T_1_-weighted images were used for the current study. The T_1_-weighted images were acquired using the parameter settings reported in detail in Supplementary Tables 2 and 3. Following MRI acquisition, the T_1_-weighted images were processed by trained and experienced neuroimaging scientists who were blinded to clinical characteristics, including group (very preterm or term-born). We used a state-of-the-art image processing pipeline, utilising recent advances in neonatal cortical surface reconstruction and parcellation. Neonatal T_1_-weighted images underwent tissue segmentation using the Infant Brain Extraction and Analysis Toolbox (iBEAT) V2.0,^19,42-45^ followed by cortical surface reconstruction and parcellation using a modified version of the surface-based Melbourne Children’s Regional Infant Brain atlas (M-CRIB-S) pipeline tuned for T_1_-weighted image contrast.^20^ The 7-year and 13-year T_1_-weighted images were processed using the FreeSurfer V6 standard pipeline,^21^ including tissue segmentation, cortical surface reconstruction and parcellation using the Desikan-Killiany-Tourville (DKT) atlas, which is available within FreeSurfer.^46^ We previously developed the atlas within M-CRIB-S (the M-CRIB-S-DKT atlas) that was applied to the neonatal scans to be compatible with the DKT atlas that was applied to the 7-year and 13-year scans,^20^ meaning cortical parcellation was consistent between the neonatal, 7-year and 13-year study points. Raw T_1_-weighted images and image processing outputs, including cortical surfaces and cortical parcellations, were manually, visually inspected for all participants. Images with severe movement or other imaging artefact were excluded (Supplementary Fig. 1). Manual editing was performed as required in line with FreeSurfer guidelines (Supplementary Table 4). Cortical surfaces for two representative included participants are shown in Supplementary Fig. 2, supporting the high quality of the surface-based data included in the analyses. Cortical volume, area and thickness measurements were extracted for the 62 equivalent cortical regions for each of the three study points. Cortical thickness was calculated consistently across all participants and time points, using the method used within FreeSurfer (the distance between the white matter and pial surfaces), which is also used within the M-CRIB-S pipeline.

### Neuropsychological assessments

Comprehensive neuropsychological assessments at age 13 years were conducted by trained and experienced neuropsychologists who were blinded to clinical characteristics, including group (very preterm or term-born). Language was assessed using the composite index score of the Clinical Evaluations of Language Fundamentals, Fourth Edition [CELF-4; mean = 100; standard deviation (SD) = 15].^47^ Memory and learning was assessed using the total trials 1–5 scaled score of the California Verbal Learning Test, Children’s Version (CVLT-C; mean = 50; SD = 10).^48^ These variables were dichotomized to enable comparisons between clinically meaningful subgroups, where language and memory impairment were defined as scores below −1 SD of each test’s respective mean. For reference, other neurodevelopmental outcomes also assessed included rates of IQ impairment, motor impairment, attention-deficit hyperactivity disorder (ADHD) and autism spectrum disorder (ASD). IQ was estimated using the Kaufman Brief Intelligence Test, Second Edition (KBIT2; mean = 100; SD = 15),^49^ where IQ impairment was defined as scores below −1 SD of the test’s mean. Motor ability was assessed using the total motor score from the Movement Assessment Battery for Children, Second Edition (MABC-2; mean = 10; SD = 3),^50^ where motor impairment was defined as scores below −1 SD of the test’s mean. ADHD and ASD were assessed using the Development and Well-Being Assessment (DAWBA) and the Diagnostic and Statistical Manual of Mental Disorders, Fifth Edition (DSM-5) criteria.^51,52^

### Statistical analysis

Participant characteristics were compared between groups (very preterm or term-born) using *t*-tests or chi-squared tests, for continuous variables (gestational age at birth, birth weight and age at MRI) or categorical variables (sex, major neonatal brain injury, bronchopulmonary dysplasia, and neurodevelopmental impairments), respectively. Age at MRI was treated as a continuous variable throughout all analyses.

For all aims, longitudinal cortical volume, area and thickness development over the age period between 0 and 13 years was investigated using generalized additive models.^53^ This type of model was selected because prior work has shown that cortical development is non-linear.^10,14,17,54^Supplementary Tables 5–7 provide more details on model selection. Generalized additive models can be used to analyse longitudinal data by adding random intercepts to the models, as discussed in more detail in the Supplementary material.^53^ This type of model has been used in many recent studies of longitudinal brain development.^10,14,17,54^

For Aim 1 (investigation of typical cortical development), models were based on data for the term-born group. Models included a smooth function of age at MRI and a random intercept to account for longitudinal observations.

For Aim 2 (investigation of sex differences in typical cortical development), models were based on data for the term-born group. Models included a smooth function of age at MRI, a random intercept to account for longitudinal observations, and sex. Models also included an interaction between sex and age at MRI, to investigate whether longitudinal cortical development differed between sexes.

For Aim 3 (comparison of cortical development between term-born and very preterm-born children), models were based on data for the term and very preterm groups. Models included a smooth function of age at MRI, a random intercept to account for longitudinal observations, sex, and group. Models also included an interaction between group and age at MRI, to investigate whether longitudinal cortical development differed between groups. To further investigate and confirm the results of Aim 3, we performed the following additional analyses: (i) we repeated the analysis adjusted for voxel size at the 0-year time point, to ensure group differences were not influenced by variation in voxel sizes at the 0-year time point; and (ii) we repeated the analysis adjusted for intracranial volume and body weight, to investigate the influence of these variables on the group difference results.

For Aim 4 (comparison of cortical development between children with normal and impaired 13-year language and memory functioning), models were based on data for the term and very preterm groups. Models included a smooth function of age at MRI, a random intercept to account for longitudinal observations, sex, group, and cognitive function group (normal or impaired language or memory). Models also included an interaction between cognitive function group and age at MRI, to investigate whether longitudinal cortical development differed between cognitive function groups.

We report model estimated values at multiple ages between 0 and 13 years, 95% confidence intervals and *P*-values that were false discovery rate (FDR)-corrected for multiple comparisons based on the number of cortical regions. Analyses were conducted using R software version 4.1.1, mgcv package version 1.8–38.^53^ Model implementation in the R software package is reported in the Supplementary material. In the models, we set the *k* parameter at 3 ensuring smooth, biologically plausible curves.^10,14,17,54^ Results were presented on the cortical surface using fsbrain version 0.5.0.^55^

## Results

### Participant characteristics

Of the 224 very preterm and 76 term-born participants who were originally recruited, 201 very preterm and 66 term-born participants had usable cortical surface-based data for at least one study point (any study point out of 0, 7 or 13 years), and all these participants were included in the analyses. Of the 201 very preterm participants who were included in the analyses, 56 participants contributed data from one scan, 88 participants contributed data from two scans, and 57 participants contributed data from three scans; of the 66 term-born participants who were included in the analyses, 34 participants contributed data from one scan, 18 participants contributed data from two scans and 14 participants contributed data from three scans. Characteristics of the participants and non-participants (who were recruited but not included), including perinatal characteristics and neurodevelopmental outcomes, are reported in Supplementary Table 1.

### Typical cortical development from birth to adolescence

**Figure 1:**
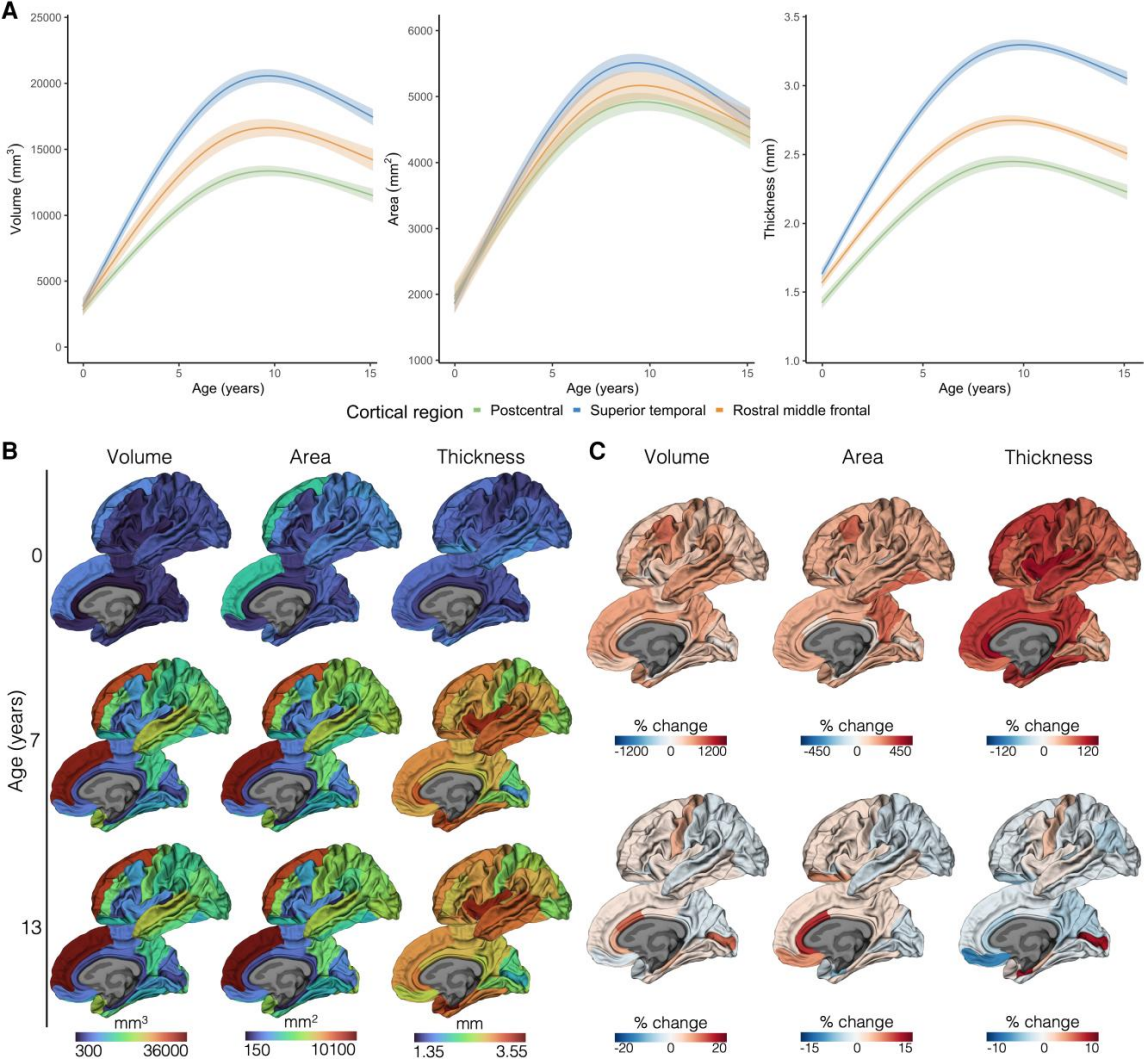
**Cortical development in term-born children**. Results are based on all term-born children who had usable data at any study point (*n* = 66; Supplementary Table 1). (**A**) The modelled longitudinal trajectories of cortical volume, area and thickness for an example sensorimotor, temporal association and prefrontal cortical region. Solid lines are the estimated values from the models and shaded ribbons are 95% confidence intervals. This demonstrates large increases in cortical volume, area and thickness in the earliest childhood years followed by comparatively more subtle changes in later years, which vary by region. (**B**) Cortical volume, area and thickness values for all cortical regions at the three study points (0, 7 and 13 years). These values were estimated from the models and mapped onto the cortical surface. This highlights regional variation in cortical metrics. (**C**) The per cent change from 0 to 7 years (*top row*) and 7 to 13 years (*bottom row*) of regional cortical volume, area and thickness, as estimated from the models and mapped onto the cortical surface. This demonstrates variation in developmental rates between regions and metrics.

We modelled longitudinal developmental trajectories of cortical volume, area and thickness in 62 cortical regions across the age period of 0 to 13 years. First, models were based on the term-born group only, to examine typical cortical development. Cortical volume, area and thickness of all cortical regions changed substantially with age in the term-born group (all FDR-corrected *P*-values < 0.05). More specifically, large increases in cortical volume, area and thickness were found in early childhood; each measure increased by an average across regions of 375%, 147% and 78%, respectively between ages 0 and 7 years. Subsequently, cortical volume, area and thickness continued to change in the later childhood years, though changes were comparatively smaller (each measure changed by an average across cortical regions of 1.8%, 1.3% and −0.4%, respectively, during ages 7 to 13 years). Some cortical regions exhibited increases and other cortical regions exhibited decreases in volume, area and thickness in this period between ages 7 and 13 years. The modelled longitudinal cortical volume, area and thickness trajectories, for example cortical regions with known different developmental timings,^56^ are shown in Fig. 1A.

In addition, we found that cortical thickness exhibited characteristic regional heterogeneity in infancy (Fig. 1B). Cortical thickness values increased in a posterior-anterior and superior-inferior sequence; lowest cortical thickness values were observed in parietal (postcentral, superior parietal, precuneus) and occipital (pericalcarine, cuneus) cortices. This regional variation in cortical thickness values persisted over childhood.

The development of cortical thickness was heterochronous (Fig. 1C). Cortical regions exhibiting the largest increases in cortical thickness over early childhood (0–7 years) were temporal (superior temporal, middle temporal, entorhinal, parahippocampal), cingulate (rostral anterior, caudal anterior, posterior and isthmus cingulate), frontal (caudal middle frontal, pars opercularis, superior frontal) and insula regions, while regions exhibiting smaller cortical thickness increases over early childhood were occipital (pericalcarine, lingual, cuneus, lateral occipital) and sensorimotor (particularly postcentral) regions. Between middle and later childhood (7–13 years), most cortical regions exhibited decreasing cortical thickness, with the greatest decreases observed in medial and lateral orbitofrontal and cingulate cortical regions. However, some regions continued to exhibit small increases in cortical thickness (≤9%), particularly the pericalcarine, entorhinal, parahippocampal, precentral, caudal middle frontal and superior temporal regions.

Cortical area displayed distinct regional developmental rates relative to cortical thickness (Fig. 1C). Regions showing the largest surface area expansions over early childhood were caudal middle frontal, precuneus, cuneus, inferior temporal and anterior cingulate cortical regions. Over middle and later childhood, many cortical regions continued exhibiting increases in area (≤13%), particularly the orbitofrontal and anterior cingulate regions, while some cortical regions showed small decreases in area (−1 to −6%), particularly the posterior occipital and parietal regions.

### Typical sex differences in cortical development

**Figure 2:**
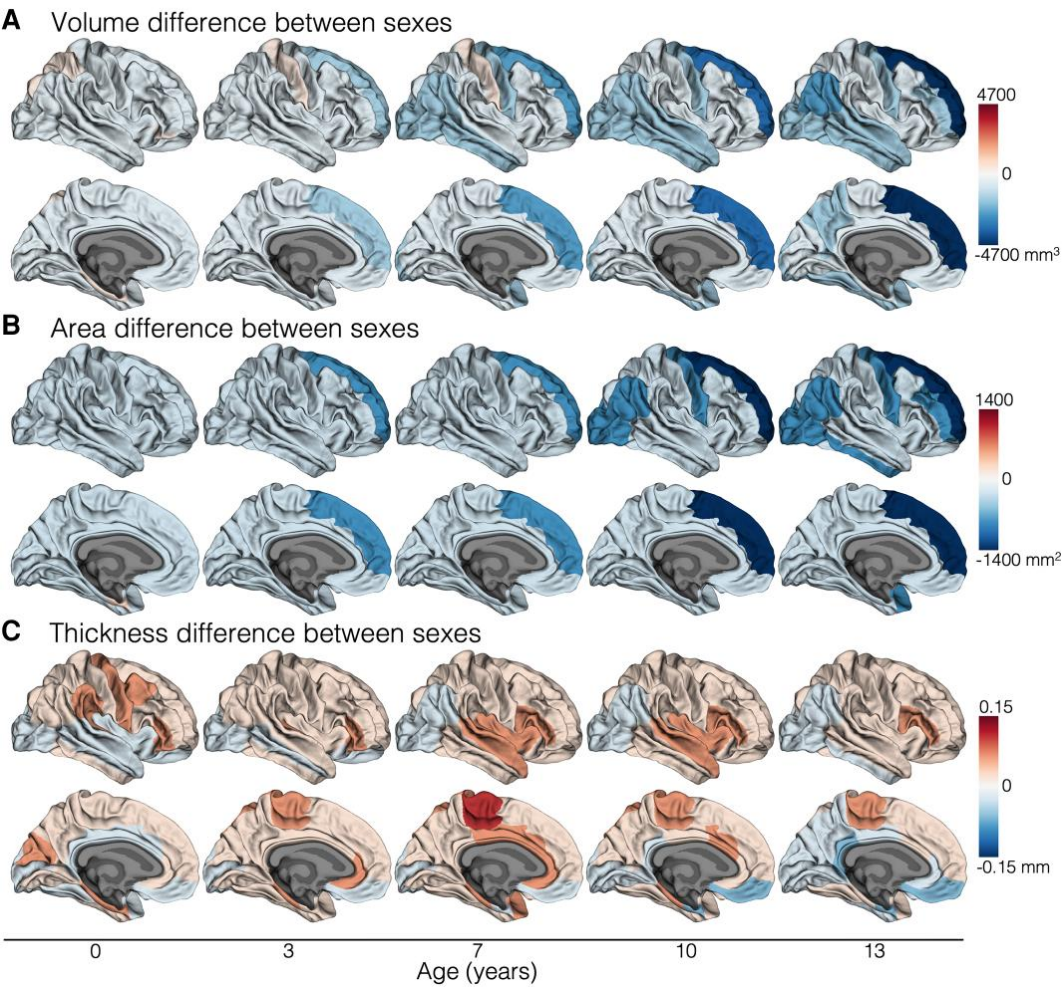
**Differences in cortical development between males and females born at term**. This figure enables visualization of cortical developmental patterns by sex across multiple cortical regions. These results are based on all term-born children who had usable data at any study point (*n* = 66; Supplementary Table 1). The magnitude of the difference in cortical volume (**A**), area (**B**) and thickness (**C**) between males and females in the term-born group at five ages (0, 3, 7, 10, 13 years) is shown plotted on the cortical surface. The sex differences at these five ages were estimated from the models (as described in the ‘Materials and methods, Statistical analysis’ section). The blue colour scale indicates females have lower values than males, while the red colour scale indicates females have higher values than males. In summary, females had lower volume and area than males in several cortical regions at age 0 years, and this volume and area reduction became further emphasized with age (shown by increasingly darker blue colours).

Typical sex differences in cortical development were observed (Fig. 2 and Supplementary Fig. 3). More specifically, longitudinal developmental trajectories during the middle and later childhood period of cortical volume and area differed between sexes in the term-born group, in several cortical regions including superior frontal, precentral, inferior parietal, inferior temporal and lateral occipital regions (FDR-corrected *P* < 0.05; Supplementary Fig. 3A). This meant there was lower cortical volume and area in females compared with males at age 0 years, which became more evident by age 13 years. This pattern of findings is shown for representative cortical regions in Supplementary Fig. 3B and C; visualization of this pattern of findings across multiple cortical regions is shown in Fig. 2. Cortical thickness developmental trajectories did not differ between sexes (FDR-corrected *P* ≥ 0.05; Supplementary Fig. 3A).

### Differences in cortical development between preterm and term-born children

**Figure 3:**
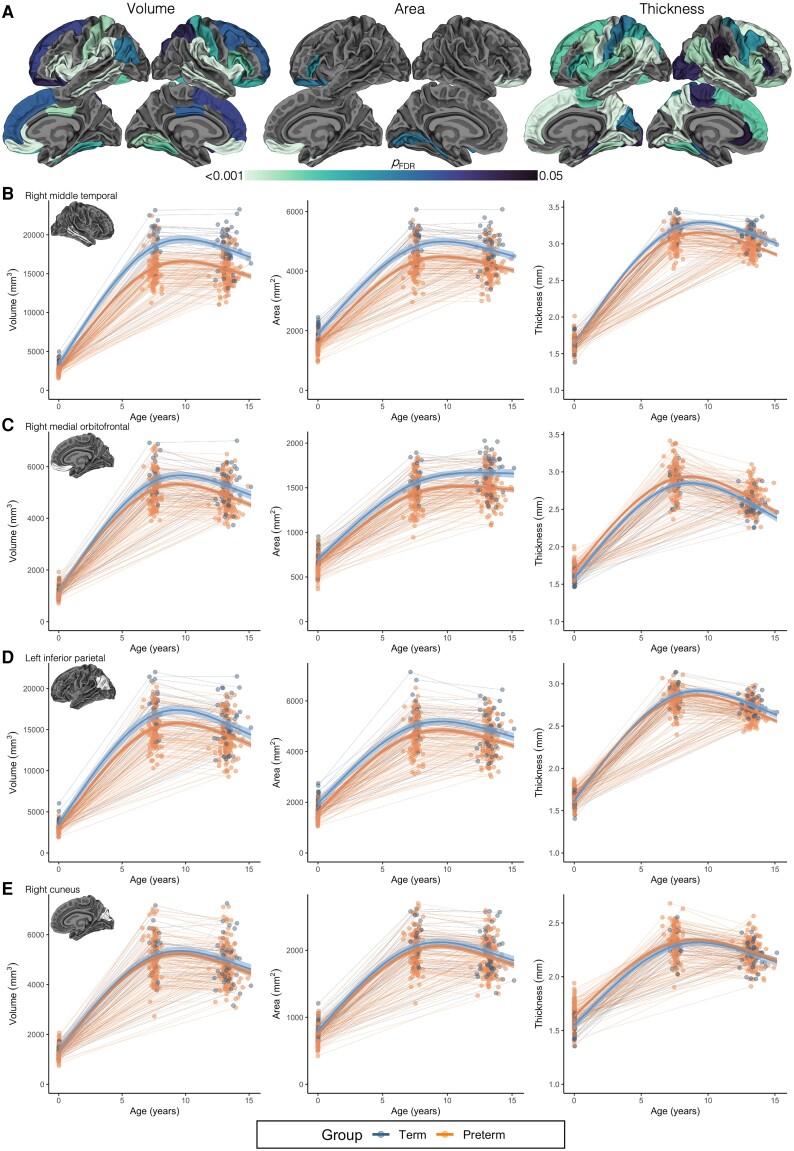
**Differences in cortical development between term-born and very preterm-born children**. These results are based on all children who had usable data at any study point (*n* = 66 term-born and *n* = 201 very preterm-born children; Supplementary Table 1). Results were adjusted for sex. (**A**) *Top row*: The cortical regions in which the longitudinal change in cortical volume, area and thickness differed between children born very preterm and children born at term [false discovery rate (FDR)-corrected *P* < 0.05]. For further interpretation of these differences shown in **A**, the plots in **B**–**E** can be referred to. (**B**–**E**) The modelled developmental trajectories of cortical volume, area and thickness by group (term = blue; very preterm = orange) for example representative temporal (**B**), frontal (**C**), parietal (**D**) and occipital (**E**) cortical regions. Thick lines with ribbons are estimated values from the models with 95% confidence intervals for each group; thin background lines and points are raw data for each participant (with some participants contributing data for 1, 2 and 3 study points). In summary, cortical volume reductions in children born preterm became more emphasized with age in temporal, frontal and parietal regions (**B**, **C** and **D**; *left*) Cortical area reductions remained static with age in most of the cortex (**B** and **D**; *middle*) except for in orbitofrontal regions where area reductions became more emphasized (**C**; *middle*). Cortical thickness developmental alterations in the preterm group were more variable; sometimes reductions became more apparent with age (temporal and parietal regions in **B** and **D**; *right*), remained static (medial orbitofrontal region in **C**; *right*) or became less apparent (occipital regions in **E**; *right*). Occipital regions mostly did not exhibit volume or area developmental alterations in the preterm group (**E**, *left* and *middle*).

**Figure 4:**
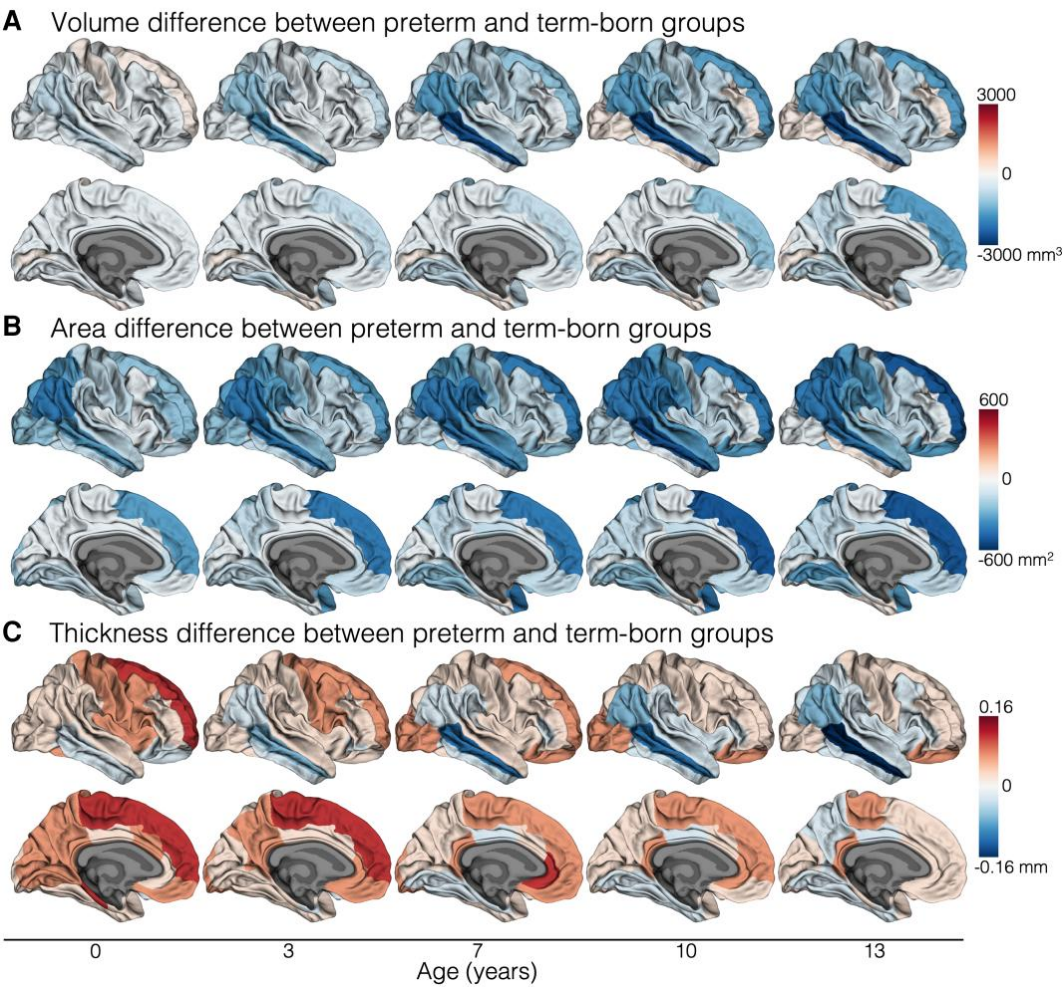
**Differences in cortical development between term-born and very preterm-born children**. This figure enables visualization of cortical developmental patterns by group across multiple cortical regions. These results are based on all children who had usable data at any study point (*n* = 66 term-born and *n* = 201 very preterm-born children; Supplementary Table 1). Results were adjusted for sex. In this figure, the magnitude of the difference in cortical volume (**A**), area (**B**) and thickness (**C**) between children born very preterm and children born at term at five ages is shown plotted on the cortical surface. The group differences at these five ages (0, 3, 7, 10, 13 years) were estimated from the statistical models (see the ‘Materials and methods, Statistical analysis’ section for more details on the modelling). The blue colour scale indicates the preterm group has lower values than the term-born group, while the red colour scale indicates the preterm group has higher values than the term-born group. In summary, children born preterm had lower volume than children born at term in many cortical regions at age 0 years, and this volume reduction became more emphasized with age (shown by increasingly darker blue colours). Children born preterm had lower area than term-born children in many cortical regions at age 0 years, and this area reduction remained relatively stable across the age period. Cortical thickness differences were more complex; the most prominent finding was that children born preterm had lower thickness in temporal regions at age 0 years than term-born children, which became more emphasized with age (shown by increasingly darker blue colours).

Longitudinal developmental trajectories of cortical volume differed between term-born and very preterm-born children in many cortical regions, particularly temporal (middle temporal), parietal (supramarginal) and frontal (pars opercularis, pars triangularis, medial orbitofrontal) regions, as well as rostral anterior cingulate and insula regions (FDR-corrected *P* < 0.05; Fig. 3A). More specifically, this meant children born very preterm exhibited reduced cortical volume at age 0 years and also lower rates of increases in cortical volume in these regions with age, particularly over early childhood (ages 0–7 years), resulting in a greater magnitude of volume reductions in these regions by ages 7 and 13 years. This pattern of volumetric development in children born very preterm compared with children born at term is shown for two representative regions (middle temporal and medial orbitofrontal) in Fig. 3B and C, left column. Cortical volume trajectories by group for all regions are displayed in Fig. 4A and Supplementary Figs 4 and 5.

Longitudinal cortical area trajectories in most regions did not differ between term-born and very preterm-born children (FDR-corrected *P* ≥ 0.05; Fig. 3A). This indicated that cortical area reductions in children born very preterm compared with children born at term were generally established by age 0 years and persisted to a similar degree across the age period. However, cortical area reductions specifically in the inferior, orbital frontal regions increased in magnitude, particularly over the early childhood years, for children born very preterm compared with children born at term (shown for the medial orbitofrontal region in Fig. 3C, middle column). Cortical area trajectories by group for all regions are displayed in Fig. 4B and Supplementary Figs 6 and 7.

In contrast to cortical area, cortical thickness trajectories differed between term-born and very preterm-born children in many cortical regions located across all lobes (FDR-corrected *P* <0.05; Fig. 3A). In many regions, children born very preterm exhibited higher cortical thickness at age 0 years but smaller increases in cortical thickness particularly in the early childhood years (ages 0–7 years), leading to similar or reduced thickness compared with term-born children in these regions by ages 7 and 13 years (including lingual, cuneus, precuneus, inferior parietal, fusiform, precentral, pars opercularis, pars triangularis and superior frontal regions; as shown for the inferior parietal and cuneus regions in Fig. 3D and E, right column). In the middle temporal region specifically, children born very preterm exhibited lower cortical thickness at age 0 years and smaller increases in cortical thickness throughout development, particularly in the early childhood years, leading to a large reduction in cortical thickness in this region by childhood and adolescence compared with term-born children (Fig. 3B, right column). Cortical thickness trajectories by group for all regions are displayed in Fig. 4C and Supplementary Figs 8 and 9.

The group differences in cortical developmental trajectories were similar after adjusting for voxel size at the 0-year time point (Supplementary Fig. 10). The group differences in cortical developmental trajectories were altered somewhat after adjusting for intracranial volume, as described in the Supplementary Fig. 11. However, the group differences in cortical developmental trajectories were little altered after adjusting for body weight (Supplementary Fig. 12).

### Differences in cortical development between children with normal and impaired cognitive functioning

**Figure 5:**
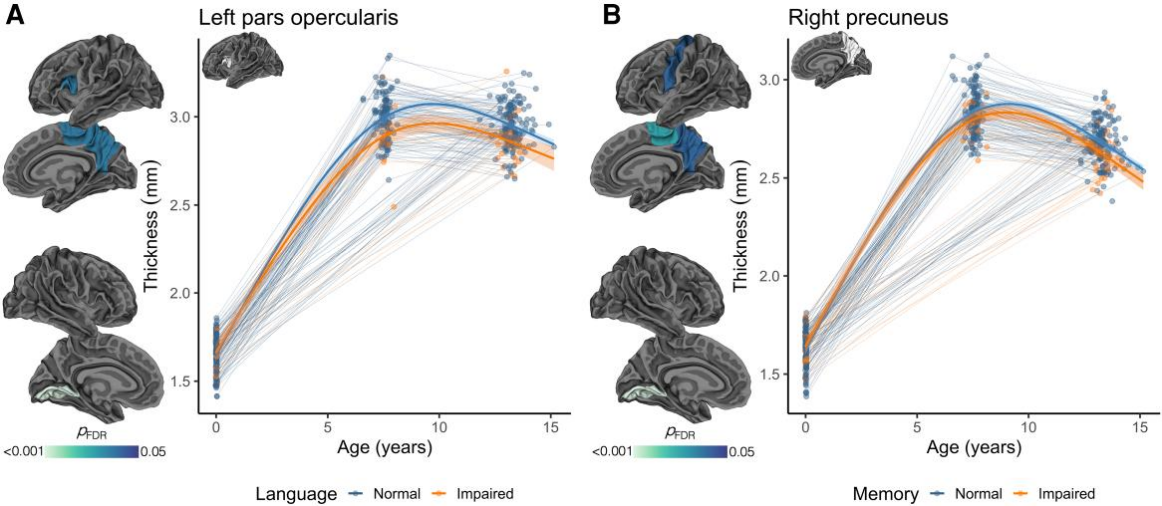
**Differences in longitudinal cortical thickness development between children with normal and impaired 13-year cognitive functioning.** (**A**) Language; (**B**) memory. Results are based on all children who had usable data at any study point (*n* = 66 term-born and *n* = 201 very preterm-born children; Supplementary Table 1). Results were adjusted for sex and group (preterm or term-born). Within each figure, the *left column* shows the cortical regions in which longitudinal cortical thickness developmental trajectories differed between children with normal and impaired 13-year cognitive functions [*P* < 0.05, false discovery rate (FDR)-corrected]. The plots show the modelled trajectories by cognitive function group (normal = blue; impaired = orange) for cortical thickness in example cortical regions. Thick lines with ribbons are estimated values from the models with 95% confidence intervals for each cognitive function group (normal or impaired); thin background lines and points are raw data for each participant (with some participants contributing data for 1, 2 and 3 study points).

Longitudinal cortical thickness trajectories in some cortical regions differed between children with normal and impaired 13-year language and memory functioning (FDR-corrected *P* < 0.05; Fig. 5). More specifically, smaller increases in cortical thickness over early childhood, and larger decreases in cortical thickness over later childhood, in the pars opercularis, precuneus, paracentral and lingual regions were found in children with impaired language compared with children with normal language (Fig. 5A). Smaller increases in cortical thickness over early childhood, and larger decreases in cortical thickness over later childhood, in the precuneus, precentral, paracentral and lingual regions were found in children with impaired memory compared with children with normal memory (Fig. 5B). Longitudinal cortical volume and area trajectories were generally similar between children with normal and impaired 13-year language and memory function (FDR-corrected *P* ≥ 0.05; except for one region, the left pars opercularis, where longitudinal cortical volume trajectories differed between children with normal and impaired 13-year language).

## Discussion

In summary, there were expected large increases in cortical volume, area and thickness over the early childhood years, followed by ongoing but comparatively more subtle changes in cortical volume, area and thickness in later childhood years in healthy term-born children. There were also expected sex differences in cortical development. Children born very preterm exhibited smaller cortical volume and area and regional increases and decreases in cortical thickness at age 0 years compared with term-born children. Additionally, children born very preterm exhibited altered longitudinal cortical developmental trajectories, resulting in amplified cortical alterations in adolescence in this group. Furthermore, children with impaired language and memory function exhibited different longitudinal cortical thickness developmental trajectories compared with children who had normal language and memory function.

Our observed typical cortical developmental patterns are consistent with current knowledge of cortical development based on recent, large-scale, cross-sectional MRI studies^14^ and longitudinal MRI studies in specific age windows.^5,6^ The neurobiological changes underlying these changes in cortical morphology metrics are under active investigation. Large increases in cortical thickness and area in the early years of life are thought to parallel complex local microstructural and cellular processes including increases in dendritic arborization, synaptogenesis, gliogenesis, axonal growth and pericortical myelination.^6,15,57^ Over later childhood and adolescence, subtle cortical thickness decreases and cortical surface area expansions parallel reorganization of dendritic arbour and increases in pericortical myelination.^6,58^ Our findings on typical sex differences in cortical development are also consistent with several prior studies, which found comparable results to the current study; that is, males have larger longitudinal cortical volume and area increases than females in infancy^5,15^ and childhood and adolescence.^59,60^ In our study, absolute values and developmental tempos of cortical thickness did not vary by sex, in line with past research.^6^

Infants born very preterm already exhibited altered cortical structure by term-equivalent age, as expected based on prior research.^27-30,61^ The current study revealed infants born very preterm also exhibited altered cortical development after term-equivalent age. Low cortical volume in very preterm-born infants became further exaggerated with age, particularly between 0 and 7 years. A key pattern emerged where in lateral temporal and parietal regions, exaggerated low volume was driven by increasingly reduced cortical thickness (with persistently low area). Conversely, in inferior, orbitofrontal regions, exaggerated low volume was due to increasingly lower cortical area (with persistently higher thickness). By age 13, very preterm-born adolescents exhibited lower volume and area in frontal, temporal and parietal regions, lower thickness in temporal regions, and higher thickness in orbitofrontal regions, compared with term-born adolescents; consistent with prior cross-sectional studies of other cohorts.^31,32^ Thus, cortical regions most vulnerable after very preterm birth are higher-order frontal, temporal and parietal regions. This may relate to the relative immaturity of these regions during the third trimester of gestation,^56^ when very preterm infants are exposed to environmental factors in the hospital.^62^ Ongoing work is needed to more fully understand the underlying cellular mechanisms contributing to observed MRI alterations; e.g. by correlating MRI measures with histology. It remains to be determined whether different alterations in temporal regions (reduced thickness) compared with orbitofrontal regions (increased thickness) in children born very preterm could point to different neurobiological alterations.

There have been few longitudinal MRI studies in preterm children. Our findings build on prior studies in the same cohort, which used voxel-based rather than surface-based neonatal MRI analysis techniques to find that low brain volume in very preterm-born infants becomes more exaggerated between ages 0 and 7 years,^33^ particularly in temporal regions,^34^ after which longitudinal brain volumetric trajectories are more similar between very preterm-born and term-born children from ages 7–13 years.^34^ Here, by fractionating cortical volume changes into cortical area and thickness changes, we provide an explanation for our prior cortical volume findings: that increasingly lower volume in temporal regions^34^ is due to increasingly lower cortical thickness. The combination of cortical area and thickness was also more sensitive than cortical volume for detecting alterations in cortical maturation associated with prematurity. Other prior longitudinal studies in different cohorts with two scans between ages 8 and 9 years^32^ or ages 15 and 20 years^31,39^ have found that longitudinal cortical volume, area and thickness trajectories did not differ between preterm and term-born children (no significant interactions between group and age). These prior findings appear broadly consistent with the findings in the current study, where longitudinal cortical developmental deviations in very preterm-born children occurred most noticeably over the earliest childhood years (ages 0–7 years) rather than during the later childhood-adolescence period (ages 7–13 years). However, we also observed that longitudinal trajectories differed, on a smaller scale than earlier time points, between ages 7 and 13 years for very preterm children compared with term-born children in some cortical regions; including temporal and parietal regions, in which very preterm children exhibited somewhat larger decreases in cortical thickness over ages 7–13 years. This shares similarities with another recent study, which acquired up to three scans between ages 4 and 12 years, and found that cortical thickness decreased significantly more over this age period in preterm-born children than term-born children in parts of the temporal and occipital cortices.^38^ Interestingly, some similar patterns of deviations from typical cortical developmental trajectories have also previously been observed in neurodevelopmental disorders, such as ADHD and ASD.^10^

Longitudinal cortical thickness development was altered in children who had impaired language and memory function compared with children who had normal language and memory function, irrespective of group (very preterm or term-born). This suggests there is a relationship between cognitive function and cortical development, and this relationship is important for all children, consistent with prior research.^10^ Cortical regions that differed between children with impaired and normal language and memory were predominantly inferior frontal and medial parietal regions, which have previously been related to these functions (e.g. the pars opercularis corresponding to Broca’s area differed between those with impaired and normal language).^63^ This analysis identified differences in cortical development between children with impaired and normal language and memory function, but no conclusions on causality can be drawn from this. The relationship between cognitive and cortical development is complex and potentially bi-directional (engagement in cognitive tasks may affect cortical maturation and vice versa). The direction cannot be determined from this analysis, as is the case for other studies in this field.^10^

Strengths of this study are the unique longitudinal cohort spanning infancy to adolescence, which included both very preterm and term-born children, collection of a wealth of perinatal, neuroimaging and neuropsychological data, and use of advanced neonatal cortical surface-based MRI analyses to breakdown cortical volume into area and thickness. Some limitations should be considered. Our cohort was recruited ∼20 years ago in the early 2000s, since when there have been updates in neonatal intensive care and neonatal neuroimaging acquisition techniques, meaning ongoing studies with more recently recruited cohorts would be valuable. Additionally, the cohort was assessed at three study points, with relatively narrow age ranges at each MRI scan, and relatively wide intervals between MRI scans. Prior studies have demonstrated that cortical thickness peaks between age 1 and 2 years^16^ and there is marked variation across cortical regions in the age at which cortical metrics peak.^14,16^ While we report later peaks in cortical thickness, and less apparent variability in peaks across cortical regions, we note that we are not able to determine the precise non-linear patterns between scans or exact ages of peaks in cortical metrics, due to the design of our longitudinal study, in which age was not densely sampled. Nevertheless, our reported typical cortical developmental patterns are broadly in line with those reported based on other typically developing cohorts where age was more densely sampled.^5,6^ While the sample size was large for this type of unique longitudinal study, we were not able to acquire usable MRI data at all three time points from all participants. To account for this, we employed statistical models designed to accommodate missing data and maximize our available sample size for our analyses. There were upgrades in scanner hardware and acquisition sequences between study points, which is an inherent challenge with longitudinal studies of this large scale and duration. Prior studies suggest that such scanning changes may introduce variation into results.^64-66^ We did not have sufficient data to assess the potential effects of scanner and sequence changes over time on our findings, e.g. through a test-retest reliability analysis. Existing post-acquisition image harmonization methods to correct for such scanning changes over time are currently challenging to apply to this type of longitudinal study design; additional work to address this in future studies would be beneficial. There were also variations across participants in voxel size at age 0 years; however, additional analyses adjusting for 0-year voxel size suggest this variation is unlikely to have influenced the results. Interindividual variability in cortical folding is an important consideration in developmental imaging studies.^67^ Using our validated neonatal parcellation pipeline,^20^ it was possible to identify the main gyri and sulci at term-equivalent age in our study, both for those born preterm and at term, and provide consistent parcellation with later time points. Visual quality assessment confirmed that our surface-based processing pipelines performed consistently across the preterm and term-born groups in our study.

There are also important future directions. Several biological and social variables may influence neurodevelopment in preterm children^68,69^; future investigation of potential independent or cumulative effects of these variables on cortical development would be important. We focused on defining absolute cortical development and we acknowledge that there is a complex interplay between cortical development, total brain development and sex effects, with different cortical regions and metrics potentially scaling differently with overall brain size as the brain grows.^10^ While we provided some preliminary data on the effects of intracranial volume and body weight on our results in the Supplementary material, additional focused studies dedicated to detailing the alternative research question of proportional cortical development to overall brain size or body size would be worthwhile in future.

In conclusion, this study demonstrates the long-lasting influence that early life experiences and stressors, such as those associated with very preterm birth, have on cortical development. In the context of broader research suggesting early life stress may accelerate brain ageing later in life,^70^ additional research is urgently needed to investigate whether very preterm birth and associated cortical dysmaturation may set a baseline for early brain ageing and cognitive decline later in life, as suggested by emerging research.^71,72^ By increasing focus on the earliest stages of neurodevelopment, we may be able to prevent adverse neurological outcomes across the lifespan.

## Supplementary Material

awad348_Supplementary_Data

## Data Availability

The datasets generated during and/or analysed during the current study are available from the corresponding author on reasonable request. The MRI data are not publicly available due to ethical restrictions. Code is provided in the Supplementary material.
