# Supplementary material for: Cortical growth from infancy to adolescence in preterm and term-born children
Source: Brain. 2023 Oct 10;147(4):1526–38. doi: 10.1093/brain/awad348 (PMC10994536; doi:10.1093/brain/awad348)
Supplement: awad348_Supplementary_Data [file awad348_supplementary_data.pdf]

Supplementary Material for

Cortical growth from infancy to adolescence in preterm and term-born children

Claire E. Kelly,[1,2,3] Deanne K. Thompson,[1,2,3,4] Chris L. Adamson,[3] Gareth Ball,[3,4] Thijs Dhollander,[3] Richard Beare,[3,5] Lillian G. Matthews,[1,2,6] Bonnie Alexander,[3,7] Jeanie L.Y. Cheong,[2,4,8,9] Lex W. Doyle,[2,8,9] Peter J. Anderson,[1,2] Terrie E. Inder[10,11]

1. Turner Institute for Brain and Mental Health, School of Psychological Sciences, Monash University, Melbourne, VIC 3800 Australia.
2. Victorian Infant Brain Studies (VIBeS), Murdoch Children's Research Institute, Melbourne, VIC 3052 Australia.
3. Developmental Imaging, Murdoch Children's Research Institute, Melbourne, VIC 3052 Australia.
4. Department of Paediatrics, The University of Melbourne, Melbourne, VIC 3052 Australia.
5. National Centre for Healthy Ageing and Peninsula Clinical School, Faculty of Medicine, Monash University, Melbourne, VIC 3199 Australia.
6. Department of Pediatric Newborn Medicine, Brigham and Women’s Hospital, Harvard Medical School, Boston, MA 02115 USA.
7. Department of Neurosurgery, The Royal Children’s Hospital, Melbourne, VIC 3052 Australia.
8. Newborn Research, The Royal Women’s Hospital, Melbourne, VIC 3052 Australia.
9. Department of Obstetrics and Gynaecology, The University of Melbourne, Melbourne, VIC 3052 Australia.
10. Center for Neonatal Research, Children’s Hospital of Orange County, Orange, CA 92868, USA
11. Department of Pediatrics, University of California, Irvine, Irvine, CA 92697, USA

Correspondence to: Claire E Kelly
Turner Institute for Brain and Mental Health, School of Psychological Sciences, 18 Innovation Walk, Monash University, Clayton, VIC, 3800, AUS.
claire.kelly@monash.edu

This PDF file includes:

- Supplementary Text
- Figs. S1 to S12
- Tables S1 to S7
- References

Supplementary methods and results: Participant numbers and characteristics
This section describes the participants involved in the longitudinal Victorian Infant Brain Study (VIBeS). A total of 224 preterm infants and 76 term-born infants were recruited into the VIBeS study. The VIBeS study involved distinct sub-studies (follow-ups) when participants were aged approximately 0, 7 and 13 years (referred to as the 'study points'). Participant numbers at each study point are described below, and are visually summarised in Fig S1.

0-year study point (term-equivalent age). Of the recruited participants, 223 preterm and 45 term-born infants underwent MRI at approximately term-equivalent age. Of those scanned, cortical surface-based data could be analysed for 143 preterm infants and 36 term-born infants. Exclusions were due to scanning occurring outside 38-42 weeks' postmenstrual age (n=9 preterm, 1 term-born infant), or poor image quality resulting from movement or other artefacts (n=71 preterm, 8 term-born infants).

7-year study point. Of the recruited participants, 197 preterm and 69 term-born children were followed up at 7 years of age. The main reasons for loss to follow-up were families declining or withdrawing from the study, living in other countries, or not being contactable. Of those followed-up, 159 preterm and 35 term-born children underwent MRI, of which cortical surface-based data could be analysed for 120 preterm and 29 term-born children. Exclusions (n=39 preterm, 6 term-born children) were due to poor image quality resulting from movement or other artefacts.

13-year study point. Of the recruited participants, 179 preterm and 61 term-born children were followed up at 13 years of age. The main reasons for loss to follow-up were families declining or withdrawing from the study, living in other countries, or not being contactable. Of those followed-up, 141 preterm and 47 term-born children underwent MRI, of which cortical surface-based data could be analysed for 140 preterm and 47 term-born children. Exclusions (n=1 preterm child) were due to poor image quality resulting from movement or other artefacts.

Included participants with data at any study point. In all, 201 preterm and 66 term-born participants had usable cortical surface-based data for at least one study point (any study point out of 0, 7 or 13 years), and all these children were included in the analyses reported in the main text of the current study. Characteristics of the 201 preterm and 66 term-born participants included in the analyses are shown in Table S1. By study design, gestational age (GA) at birth and birth weight were lower in the preterm group than the term-born group. Age and body weight were slightly lower in the preterm group than the term-born group at the 0-year study point, but age and body weight did not differ between preterm and term-born groups at the 7-year and 13-year study points. Sex did not differ between the preterm and term-born groups. Intracranial volume was lower in the preterm group than the term-born group at all study points, though this difference reached significance at the 7-year and 13-year timepoints only. As was expected based on prior research, including prior research conducted on the current cohort,[73,74] rates of language and memory impairment at age 13 years were higher in the preterm group than the term-born group (all $p<0.05$). Regarding other neurodevelopmental outcomes, rates of IQ and motor impairment at age 13 years were higher in the preterm group than the term-born group ($p<0.05$). Rates of attention-deficit/hyperactivity disorder (ADHD) and autism spectrum disorder (ASD) were low and similar between the preterm and term-born groups ($p>0.05$).

Included participants with longitudinal data at two or three study points. For the 201 preterm and 66 term-born participants who had usable cortical surface-based data at any study point, a breakdown of the number of these participants who had usable cortical surface-based data at 1, 2 or 3 study points, and their baseline perinatal characteristics, is shown in Table S1. These perinatal characteristics (GA at birth, birth weight, sex, major brain injury and bronchopulmonary dysplasia) did not differ between participants who had usable data at 1, 2 and 3 study points (all $p>0.05$). This suggests there was no substantial difference in key, clinically important perinatal characteristics between the children who contributed 1, 2 and 3 scans.

Included participants compared with non-participants. Baseline perinatal characteristics (GA at birth, birth weight, sex, major brain injury and bronchopulmonary dysplasia) did not differ between participants included in the analyses (201 preterm, 66 term) and the remaining non-participants who were recruited but not included in the analysis (all $p>0.05$), except included term-born participants had a slightly younger GA at birth than term-born non-participants (mean (SD): 39.1 (1.3) vs. 40.0 (1.0), $p=0.03$). This suggests included participants were generally representative of the total cohort.

Sample limitations and strategies to address these limitations. As described above, sample size and follow-up rates in the VIBeS study were relatively high for this type of longitudinal cohort study, which was challenging and time-consuming to carry out. However, we acknowledge there was some loss to follow-up which resulted in our final dataset containing some missing data. The statistical models used were able to handle all available data, which helped to maximise sample size and statistical precision, and reduce any potential bias due to attrition.[75]

We also acknowledge the relatively smaller sample size of the term-born group. Nevertheless, our analysis of typical cortical development and typical sex differences in cortical development in the term-born group produced results that are consistent with knowledge generated from prior studies using larger typically developing samples, as discussed in other sections.

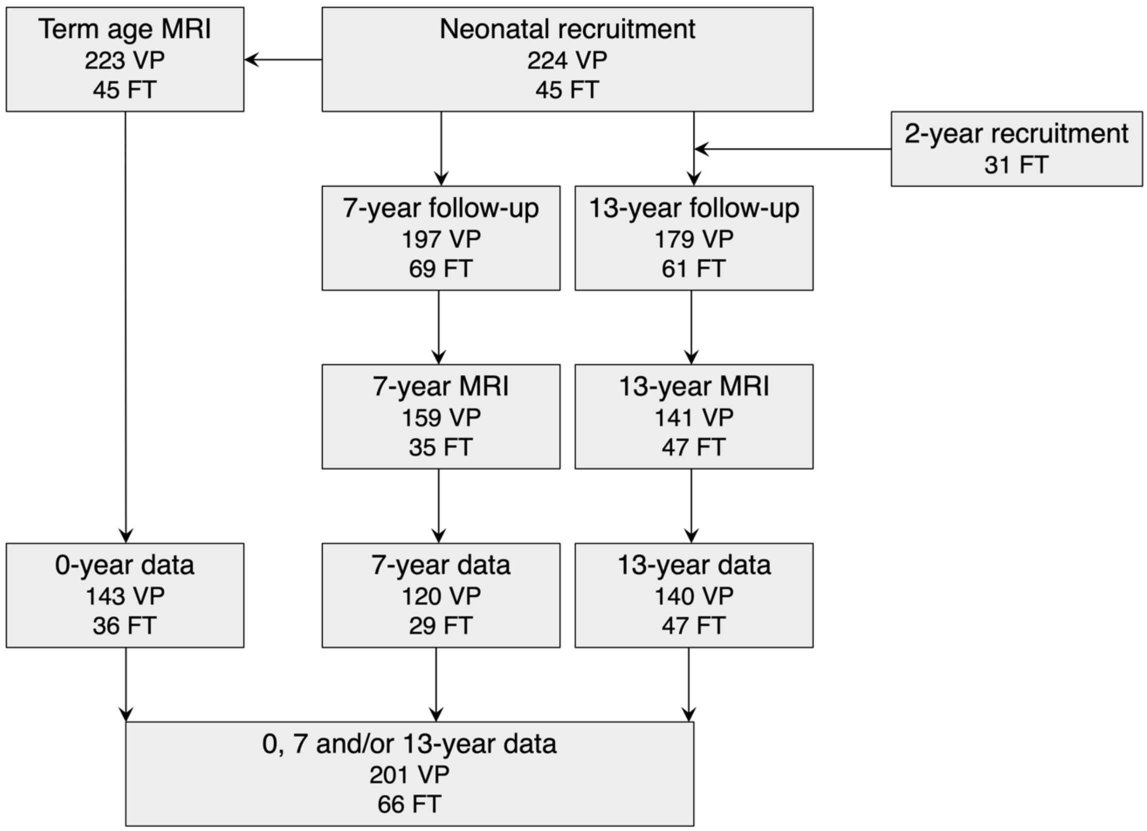

Fig. S1. Participant flowchart. FT: full-term; MRI: magnetic resonance imaging; VP: very preterm. 'Data' refers to cortical surface-based data (cortical volume, area and thickness in cortical regions) that was of sufficient quality for inclusion in the analyses.

Table S1. Participant numbers and characteristics.

	Data for any study point		Data for 1 study point		Data for 2 study points		Data for 3 study points	
	**Preterm N=201**	**Term N=66**	**Preterm N=56**	**Term N=34**	**Preterm N=88**	**Term N=18**	**Preterm N=57**	**Term N=14**
**GA at birth (weeks)**	27.5 (1.9)	39.1 (1.3)	27.2 (1.9)	39.1 (1.4)	27.7 (1.9)	39.0 (1.2)	27.5 (1.9)	38.9 (1.2)
**Birth weight (g)**	966 (221)	3321 (518)	928 (225)	3340 (537)	965 (223)	3255 (469)	1005 (213)	3359 (559)
**Male sex**	102 (51)	34 (52)	28 (50)	19 (56)	45 (51)	8 (44)	29 (51)	7 (50)
**Major brain injury**	13 (6)	0 (0) N: 44	3 (5)	0 (0) N:12	8 (9)	0 (0)	2 (4)	0 (0)
**BPD**	65 (32)	0 (0) N: 45	21 (38)	0 (0) N:13	30 (34)	0 (0)	14 (25)	0 (0)
**Age at 0-year MRI (years)**	0.01 (0.02) N: 143	0.02 (0.02) N: 36						
**Age at 7-year MRI (years)**	7.53 (0.26) N: 120	7.61 (0.20) N: 29						
**Age at 13-year MRI (years)**	13.30 (0.40) N: 140	13.30 (0.54) N: 47						
**Weight at 0 years (kg)**	2.94 (0.47) N: 143	3.53 (0.45) N: 35						
**Weight at 7 years (kg)**	24.89 (5.34) N: 118	26.23 (3.81) N: 29						

Table S1 (continued). Participant numbers and characteristics.

	Data for any study point		Data for 1 study point		Data for 2 study points		Data for 3 study points	
	**Preterm N=201**	**Term N=66**	**Preterm N=56**	**Term N=34**	**Preterm N=88**	**Term N=18**	**Preterm N=57**	**Term N=14**
**Weight at 13 years (kg)**	49.91 (11.72) N: 137	52.64 (12.62) N: 44						
**ICV at 0 years (cm^3)**	439 (57) N: 134	453 (54) N: 28						
**ICV at 7 years (cm^3)**	1328 (118) N: 120	1433 (111) N: 29						
**ICV at 13 years (cm^3)**	1562 (140) N: 140	1630 (158) N: 47						
**Impaired language at 13 years**	26 (17) N: 154	2 (4) N: 49						
**Impaired memory at 13 years**	37 (22) N: 169	5 (9) N: 55						
**IQ impairment at 13 years**	33 (20) N: 169	1 (2) N: 55						
**Motor impairment at 13 years**	34 (22) N: 155	3 (6) N: 51						
**ADHD at 13 years**	3 (3) N: 120	0 (0) N: 44						
**ASD at 13 years**	7 (6) N: 120	1 (2) N: 44						

Data are mean and standard deviation for continuous variables (gestational age (GA) at birth, birth weight and age at MRI), or N and % for categorical variables (sex, major brain injury, bronchopulmonary dysplasia (BPD), and neurodevelopmental impairments). Within the cells, the sample size is provided if this differed to the total sample size listed in the second row of the table. ADHD: attention-deficit/hyperactivity disorder (ADHD); ASD: autism spectrum disorder; ICV: intracranial volume.

Supplementary methods: MRI acquisition

Table S2. MRI acquisition details.

Study point	Scanner	Sequence details
0-year (TEA)	1.5 T General Electric MRI scanner (Signa LX Echospeed System; General Electric, Milwaukee, WI)	T1-weighted images were acquired with 1.5-3 mm coronal slices, flip angle 45°, TR 35 ms, TE 9 ms, FOV 210 × 157 mm, matrix 256 × 192, and in plane resolution 0.4-0.7 mm^2
7-year	3 T Siemens Trio MRI scanner (Siemens, Erlangen, Germany)	T1-weighted images were acquired with 0.8 mm isotropic voxels, TR 1900 ms, TE 2.27 ms, flip angle 9°, FOV 210 × 210 mm, and matrix 256 × 256
13-year	3 T Siemens Trio MRI scanner (Siemens, Erlangen, Germany; the same scanner as the 7-year study point)	T1-weighted images were acquired with 0.9 mm isotropic voxels, TR 2530 ms, TEs 1.77, 3.51, 5.32, 7.2 ms, flip angle 7°, FOV 230 × 209 mm, and matrix 256 × 256

TEA: term-equivalent age; TR: repetition time; TE: echo time; FOV: field of view.

Table S3. T1-weighted image voxel sizes at the 0-year study point.

	**Preterm (N:143)**	**Term (N:36)**	**p-value**
Coronal slice thickness (mm), mean (SD)	2.17 (0.80)	2.07 (0.51)	0.5
In plane resolution (mm^2), mean (SD)	0.49 (0.16)	0.47 (0.13)	0.4

SD: standard deviation.

Supplementary methods: MRI processing

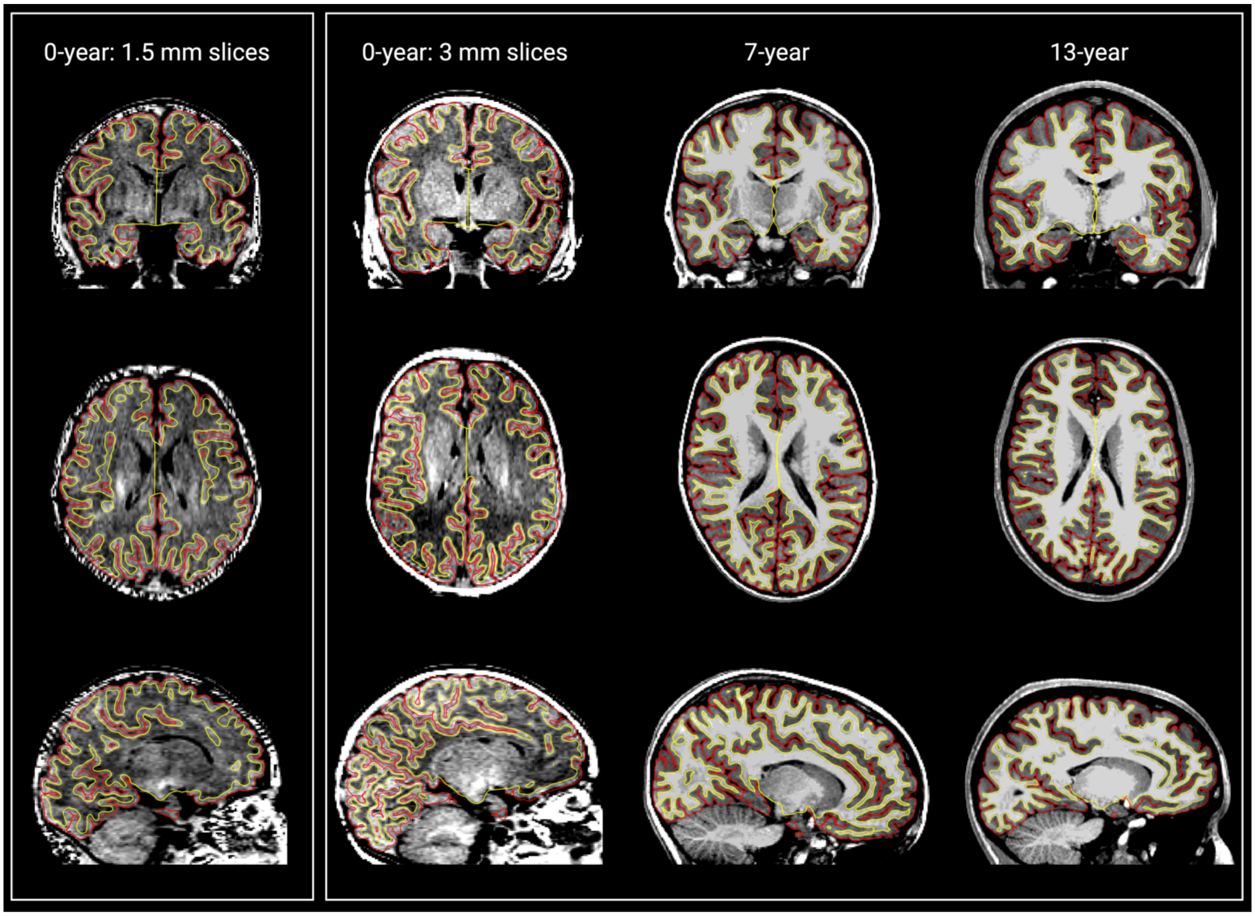

Fig. S2. Cortical surfaces across ages and 0-year voxel sizes. This figure shows the white matter (yellow) and pial (red) surfaces overlaid on T1-weighted images in all three planes (coronal, axial and sagittal) for example participants: one participant whose 0-year T1-weighted images were acquired with 1.5 mm slice thickness (1st column) and a second participant whose 0-year T1-weighted images were acquired with 3 mm slice thickness (2nd column). For the second participant, images at all three timepoints (ages 0, 7 and 13 years) are shown. This figure demonstrates the quality of the included cortical surfaces across 0-year voxel sizes and ages.

Cortical surface extraction and manual inspection and editing for the 7-year and 13-year timepoints has been described and validated in prior publications.[75-81] For the 0-year timepoint, manual inspection and editing was done using the same principles as used for the 7-year and 13-year timepoints by experienced neuroimaging scientists who were blinded to clinical characteristics, including group status. For a small number of participants at the 0-year timepoint, manual edits were done to output files (voxel segmentations) of the M-CRIB-S pipeline so that the cortical surface extractor placed surfaces in the correct positions as determined by visual quality assessment (Table S4). The manual edits performed can be broken down into three types:

1. Edits to the white matter segmentation, to extend the white matter surface further out, and in turn to extend the pial surface further out, in cases when the surfaces did not fully encapsulate the white matter and gyrus (i.e., to correct insufficient penetration of the surfaces into gyral crowns)
2. Edits to the brain mask, in cases when the pial surface was overextended outside the pial or brain
3. Edits to the white matter segmentation, to fix topology errors in the white matter surface

Table S4. Number of participants whose data had each type of manual edit for the 0-year timepoint.

	**White matter edit**	**Brain mask edit**	**Topology edit**
Preterm, N	2	3	5
Term, N	6	4	0

*Note: 2 term-born participants had both white matter edits and brain mask edits, hence the total number of participants edited is 18.

Supplementary methods: Model selection

To model longitudinal cortical development in our study, we used generalised additive models (GAMs), which have been used in many recent studies of brain development.[82-85] To ensure GAMs were appropriate to use for modelling cortical data in our dataset, we carefully specified model parameters and compared model goodness-of-fit to an alternative, linear mixed-model approach. The *k* parameter of the GAM sets the basis dimension of the smooth function, specifying the upper limit of degrees-of-freedom for the model fit. Lower numbers constrain the model, penalising overcomplicated or 'wiggly' functions that are likely to overfit the data. In this study, we set k=3 ensuring smooth, biologically plausible curves.[82-85] In line with recommendations for best practices in studies of brain structural development,[84] model fit indices, the Akaike information criterion (AIC) and Bayesian information criterion (BIC), were calculated for both the GAMs and linear mixed effects models (LMEs), which showed GAMs provided lower AIC and BIC values, and thus a better model fit, than LMEs (Tables S5-S7). Additionally, model fit indices were calculated for GAMs with random slopes added, to check the effect of adjusting for subject-specific deviations from the cortical-age relationship.[86,87] This adds additional flexibility to the models, but increases model complexity and risk of overfitting. For cortical volume and area measures, model fit was similar or improved by a small amount by adding random slopes to the GAMs. For cortical thickness measures, model fit was similar, sometimes slightly poorer, and sometimes slightly improved by adding random slopes to the GAMs (Tables S5-7). Group trajectories and the main results and conclusions of the manuscript were not altered by adding random slopes to the GAMs. GAM analyses were conducted as described in the main manuscript. LME analyses were conducted using R software version 4.1.1, nlme package version 3.1-152, lme function.

Table S5. Model selection, for models based on cortical volume.

Aim	Region	Model	AIC	BIC
1	Right superior frontal	GAM	2111.00	2234.96
		GAM-slopes	1984.13	2130.42
		LME	2284.80	2295.60
	Left inferior parietal	GAM	1969.23	2108.66
		GAM-slopes	1873.21	2019.10
		LME	2142.40	2153.20
	Right middle temporal	GAM	1939.75	2085.29
		GAM-slopes	1831.51	1980.31
		LME	2143.95	2154.75
	Left lateral occipital	GAM	1944.89	2085.65
		GAM-slopes	1857.97	2002.63
		LME	2101.10	2111.90
2	Right superior frontal	GAM	2093.02	2214.23
		GAM-slopes	1985.67	2131.80
		LME	2252.87	2268.96
	Left inferior parietal	GAM	1970.15	2111.06
		GAM-slopes	1876.78	2031.76
		LME	2118.05	2134.14
	Right middle temporal	GAM	1939.26	2086.55
		GAM-slopes	1806.81	1963.52
		LME	2117.17	2133.26
	Left lateral occipital	GAM	1943.76	2083.10
		GAM-slopes	1856.43	2003.14
		LME	2074.46	2090.56
3	Right superior frontal	GAM	9543.97	10298.31
		GAM-slopes	8997.54	9879.04
		LME	10454.74	10484.38
	Left inferior parietal	GAM	8942.79	9756.25
		GAM-slopes	8467.88	9346.79
		LME	9766.57	9796.21
	Right middle temporal	GAM	8841.32	9630.21
		GAM-slopes	8294.10	9186.85
		LME	9740.59	9770.24
	Left lateral occipital	GAM	8926.63	9693.70
		GAM-slopes	8519.14	9374.88
		LME	9683.81	9713.45
4	Right superior frontal	GAM	7738.53	8327.21
		GAM-slopes	7333.07	8041.74

		LME	8448.17	8480.32
	Left inferior parietal	GAM	7263.02	7878.64
		GAM-slopes	6944.41	7652.16
		LME	7907.46	7939.61
	Right middle temporal	GAM	7214.10	7773.68
		GAM-slopes	6804.63	7521.16
		LME	7870.72	7902.87
	Left lateral occipital	GAM	7206.98	7808.28
		GAM-slopes	6917.48	7608.85
		LME	7830.89	7863.04

In this table, data are reported for models of four representative frontal, parietal, temporal and occipital regions; model selection results were similar for the remaining 58 regions. For aim 4 specifically, data are based on models for language; model selection results were similar for the models for memory. AIC: Akaike information criterion; BIC: Bayesian information criterion; GAM: generalised additive model; GAM-slopes: GAM with the addition of random slopes; LME: linear mixed-effects model.

Table S6. Model selection, for models based on cortical area.

Aim	Region	Model	AIC	BIC
1	Right superior frontal	GAM	1802.04	1944.97
		GAM-slopes	1785.75	1916.38
		LME	1973.16	1983.96
	Left inferior parietal	GAM	1666.15	1817.50
		GAM-slopes	1659.75	1794.32
		LME	1843.57	1854.37
	Right middle temporal	GAM	1612.30	1763.83
		GAM-slopes	1592.85	1729.34
		LME	1807.43	1818.23
	Left lateral occipital	GAM	1657.78	1815.14
		GAM-slopes	1642.44	1782.27
		LME	1854.50	1865.30
2	Right superior frontal	GAM	1789.82	1931.41
		GAM-slopes	1784.27	1912.20
		LME	1941.85	1957.94
	Left inferior parietal	GAM	1666.76	1819.30
		GAM-slopes	1660.54	1803.09
		LME	1823.37	1839.46
	Right middle temporal	GAM	1605.22	1763.35
		GAM-slopes	1591.00	1732.14
		LME	1784.04	1800.13
	Left lateral occipital	GAM	1661.44	1817.81
		GAM-slopes	1640.84	1782.84
		LME	1832.41	1848.50
3	Right superior frontal	GAM	8172.79	9020.89
		GAM-slopes	7986.85	8805.49
		LME	8998.21	9027.85
	Left inferior parietal	GAM	7621.18	8504.07
		GAM-slopes	7474.90	8299.58
		LME	8418.36	8448.00
	Right middle temporal	GAM	7371.00	8225.66
		GAM-slopes	7315.01	8090.26
		LME	8221.41	8251.05
	Left lateral occipital	GAM	7706.49	8552.18
		GAM-slopes	7538.26	8351.40
		LME	8524.69	8554.33
4	Right superior frontal	GAM	6612.58	7270.69
		GAM-slopes	6488.36	7151.76

		LME	7283.99	7316.14
	Left inferior parietal	GAM	6171.81	6841.86
		GAM-slopes	6065.39	6734.02
		LME	6828.10	6860.25
	Right middle temporal	GAM	5964.54	6598.78
		GAM-slopes	5928.59	6549.42
		LME	6645.97	6678.12
	Left lateral occipital	GAM	6203.78	6859.84
		GAM-slopes	6115.31	6770.94
		LME	6893.57	6925.72

In this table, data are reported for models of four representative frontal, parietal, temporal and occipital regions; model selection results were similar for the remaining 58 regions. For aim 4 specifically, data are based on models for language; model selection results were similar for the models for memory. AIC: Akaike information criterion; BIC: Bayesian information criterion; GAM: generalised additive model; GAM-slopes: GAM with the addition of random slopes; LME: linear mixed-effects model.

Table S7. Model selection, for models based on cortical thickness.

Aim	Region	Model	AIC	BIC
1	Right superior frontal	GAM	-179.08	-125.08
		GAM-slopes	-201.27	-117.46
		LME	73.43	84.23
	Left inferior parietal	GAM	-181.66	-120.04
		GAM-slopes	-189.70	-119.79
		LME	64.27	75.07
	Right middle temporal	GAM	-167.10	-78.69
		GAM-slopes	-181.91	-85.43
		LME	102.99	113.80
	Left lateral occipital	GAM	-167.00	-90.99
		GAM-slopes	-170.59	-93.56
		LME	-29.70	-18.90
2	Right superior frontal	GAM	-177.83	-120.87
		GAM-slopes	-200.41	-113.39
		LME	88.14	104.23
	Left inferior parietal	GAM	-177.98	-109.46
		GAM-slopes	-187.97	-109.45
		LME	79.33	95.42
	Right middle temporal	GAM	-164.93	-71.42
		GAM-slopes	-179.44	-78.89
		LME	117.55	133.64
	Left lateral occipital	GAM	-163.26	-84.03
		GAM-slopes	-168.16	-86.85
		LME	-12.78	3.31
3	Right superior frontal	GAM	-854.39	-308.14
		GAM-slopes	-900.47	-339.08
		LME	300.04	329.68
	Left inferior parietal	GAM	-860.98	-387.61
		GAM-slopes	-878.19	-414.33
		LME	230.02	259.66
	Right middle temporal	GAM	-790.65	-225.88
		GAM-slopes	-825.91	-268.86
		LME	394.42	424.06
	Left lateral occipital	GAM	-830.69	-172.92
		GAM-slopes	-749.82	-302.43
		LME	-67.51	-37.87
4	Right superior frontal	GAM	-681.74	-271.97
		GAM-slopes	-722.95	-260.93

		LME	243.15	275.30
	Left inferior parietal	GAM	-686.30	-320.31
		GAM-slopes	-692.41	-322.54
		LME	193.17	225.32
	Right middle temporal	GAM	-639.44	-205.92
		GAM-slopes	-674.59	-209.75
		LME	329.35	361.50
	Left lateral occipital	GAM	-701.00	-173.59
		GAM-slopes	-606.09	-242.36
		LME	-34.71	-2.57

In this table, data are reported for models of four representative frontal, parietal, temporal and occipital regions; model selection results were similar for the remaining 58 regions. For aim 4 specifically, data are based on models for language; model selection results were similar for the models for memory. AIC: Akaike information criterion; BIC: Bayesian information criterion; GAM: generalised additive model; GAM-slopes: GAM with the addition of random slopes; LME: linear mixed-effects model.

Supplementary methods: Generalised additive model implementation in the R software

This section describes the implementation of the statistical analyses reported in the main manuscript in the gam function, mgcv package, R software.[87]

Given an R dataframe with rows containing observations, and columns containing variables [cortical volume, area and thickness metrics for each cortical region, ID, age at MRI, sex, group (preterm or term), language function group (impaired or normal) and memory function group (impaired or normal)], the following model specifications (in R syntax) were used for each region and metric. For aim 1 and 2, the dataframe contained only term-born participants, while for aim 3 and 4 the dataframe contained both term and preterm participants.

Aim 1: Typical development with age in term-born children

$$y \sim s(age, k = 3) + s(ID, bs =' re')$$

Where y represents a regional volume/thickness/area, age is the participant's age, and ID is a participant specific identifier. The $'re'$ term specifies random intercepts per participant to account for longitudinal observations.

Aim 2: Differences in cortical development between term-born males and females

$$y\sim s(age, k = 3) + sex + s(age, by = sex, k = 3) + s(ID, bs =' re')$$

Where sex is an ordered factor specifying participant sex. The smooth-by-ordered-factor term specifies an interaction effect between age and sex. When using ordered factors, a reference level smooth is required, as well as a separate parametric term for the ordered factor.[87]

Aim 3: Differences in cortical development between preterm and term-born children

$$y \sim s(age, k = 3) + group + s(age, by = group, k = 3) + s(ID, bs =' re') + sex$$

where $group$ indicates preterm/term control as an ordered factor.

Aim 4: Differences in cortical development between cognitive function groups

$$y \sim s(age, k = 3) + score + s(age, by = score, k = 3) + s(ID, bs =' re') + sex + group$$

Where $score$ indicates cognitive groups (normal or impaired for language or memory) as an ordered factor.

Note that for all the above models, random slopes can be added by including: s(ID, Age, bs="re").

Supplementary results: Extended results on typical sex differences in cortical development

This section provides additional data for the investigation of sex differences in cortical development in the term-born group.

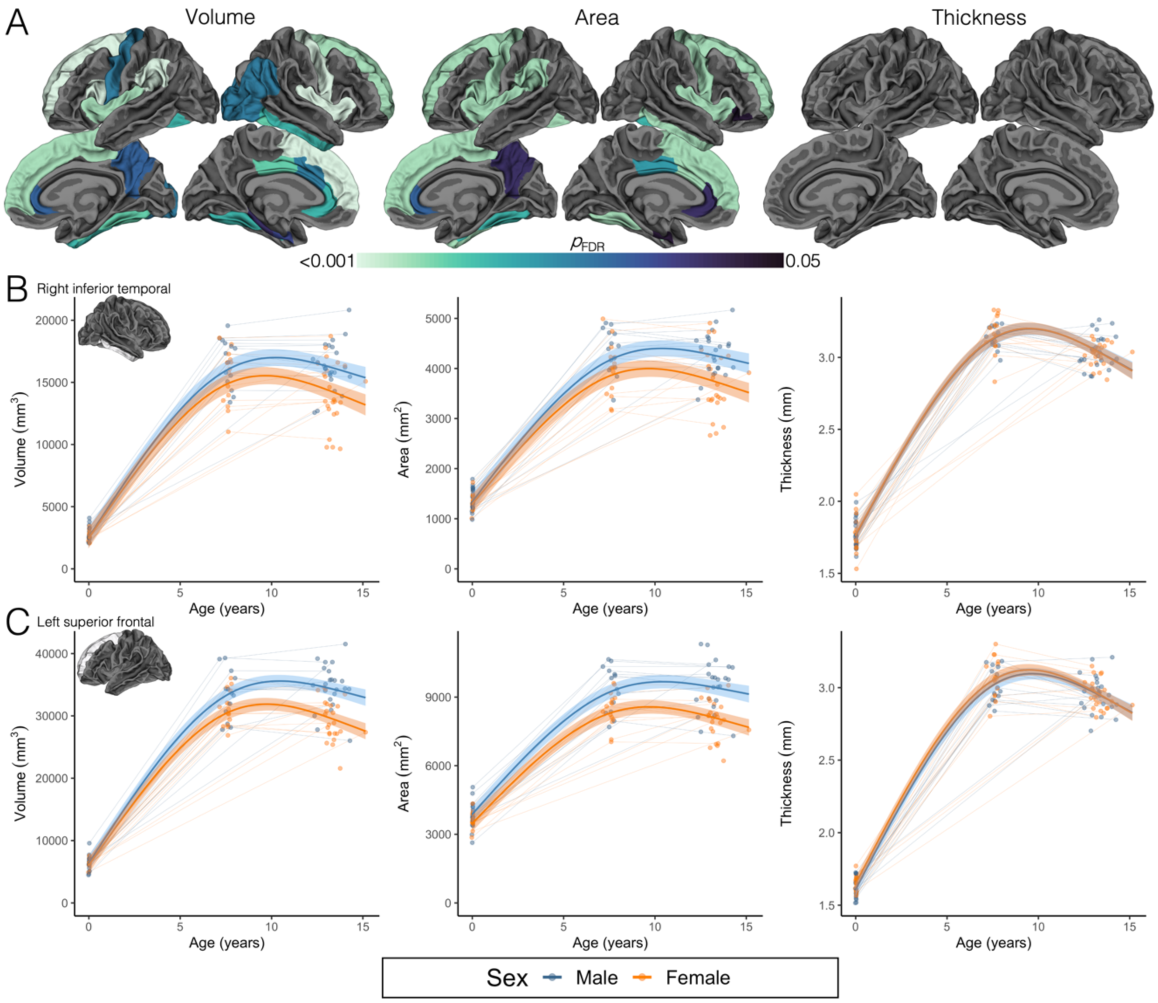

Fig. S3. Differences in cortical development between males and females born at term. These results are based on all term-born children who had usable data at any study point (n=66; Table S1). Part A (top row, left and middle columns) shows the cortical regions in which longitudinal cortical volume and area developmental trajectories differed between males and females ($p<0.05$, false discovery rate (FDR)-corrected). For further interpretation of these differences in part A, the plots in part B and C can be referred to. There were no differences in cortical thickness developmental trajectories between sexes (A, far right). Part B and C (middle and bottom rows) show the modelled longitudinal developmental trajectories by sex (males: blue; females: orange) for example representative cortical regions (part B: right inferior temporal; part C: left superior frontal). Thick lines with ribbons are predicted values from the models with 95% confidence intervals for each sex; thin background lines and points are raw data for each participant.

Supplementary results: Extended results on differences in cortical development between term-born and preterm-born children

The following pages show the modelled cortical volume (Fig. S4 and S5), cortical area (Fig. S6 and S7), and cortical thickness (Fig. S8 and S9) trajectories by group for all 62 cortical regions.

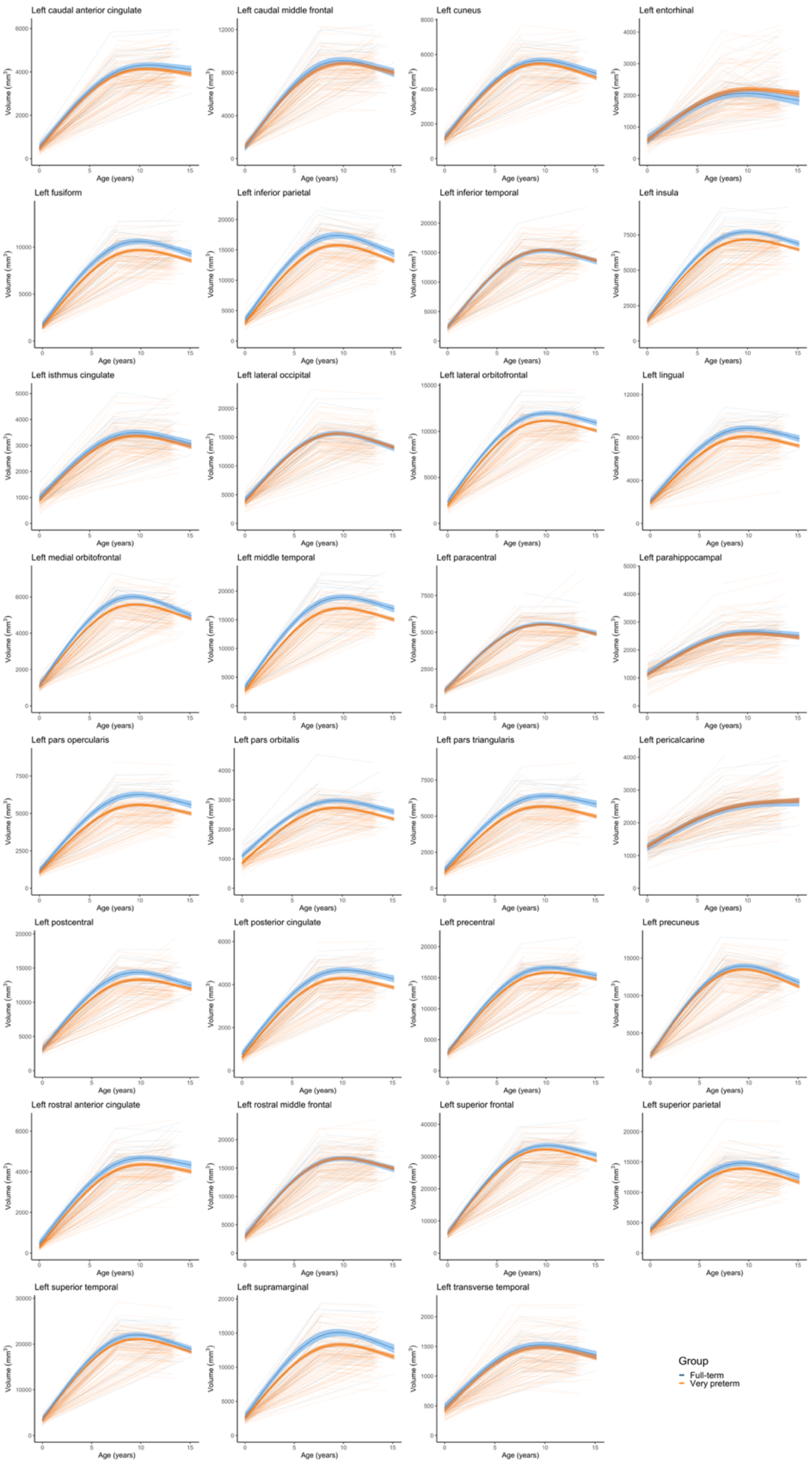

Fig. S4. Modelled trajectories by group for *volume* of *left* hemisphere cortical regions.

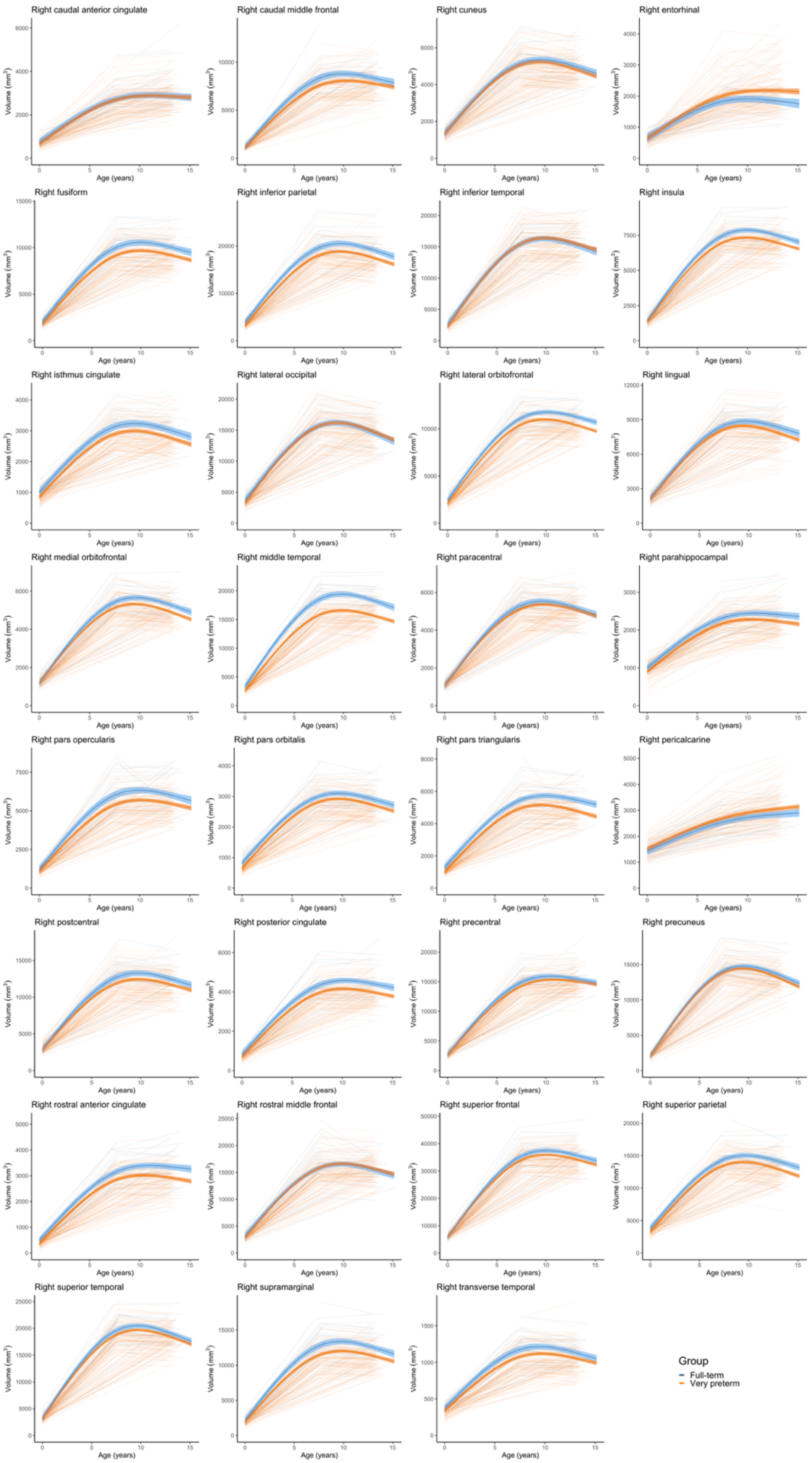

Fig. S5. Modelled trajectories by group for *volume* of *right* hemisphere cortical regions.

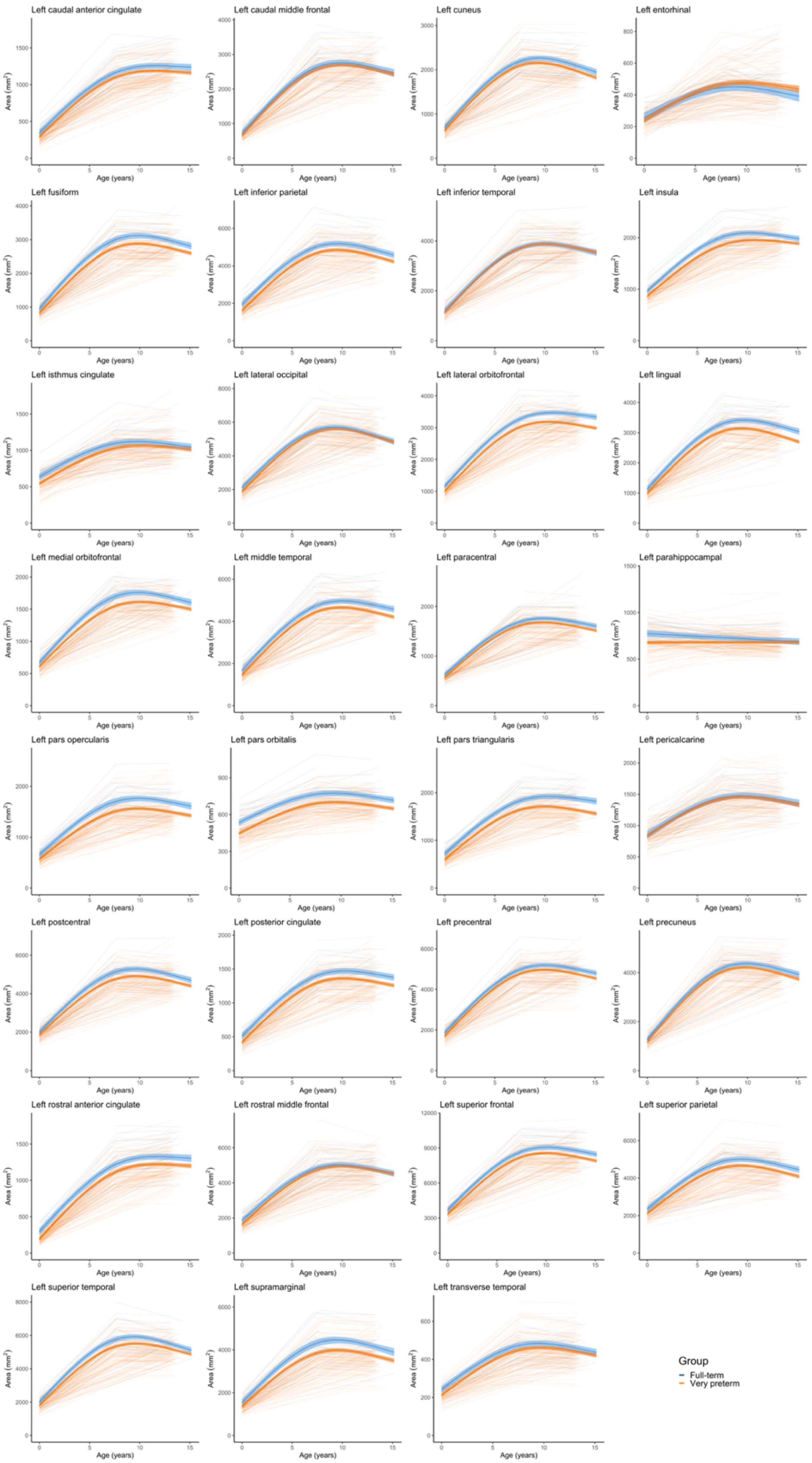

Fig. S6. Modelled trajectories by group for *area* of *left* hemisphere cortical regions.

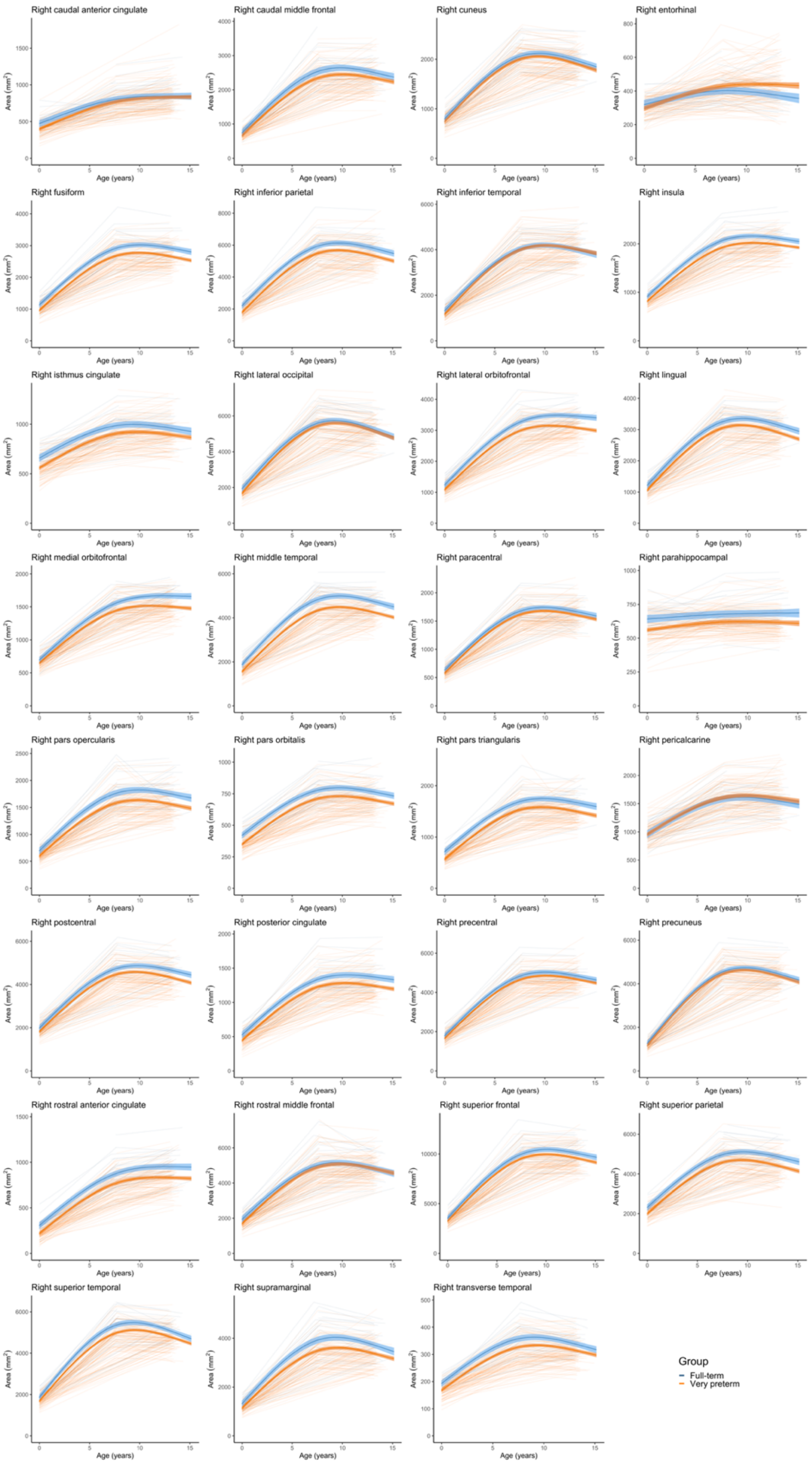

Fig. S7. Modelled trajectories by group for *area* of *right* hemisphere cortical regions.

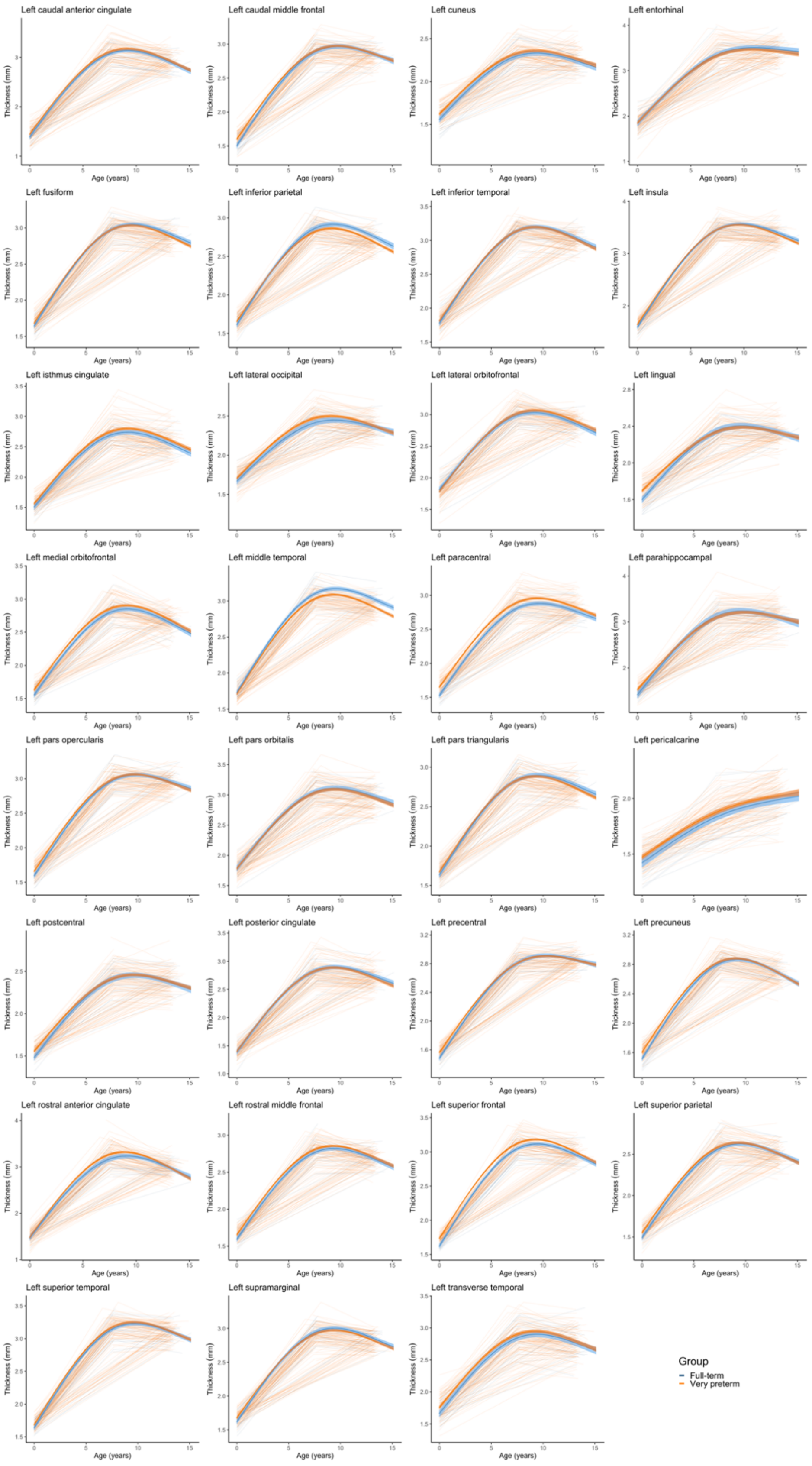

Fig. S8. Modelled trajectories by group for *thickness* of *left* hemisphere cortical regions.

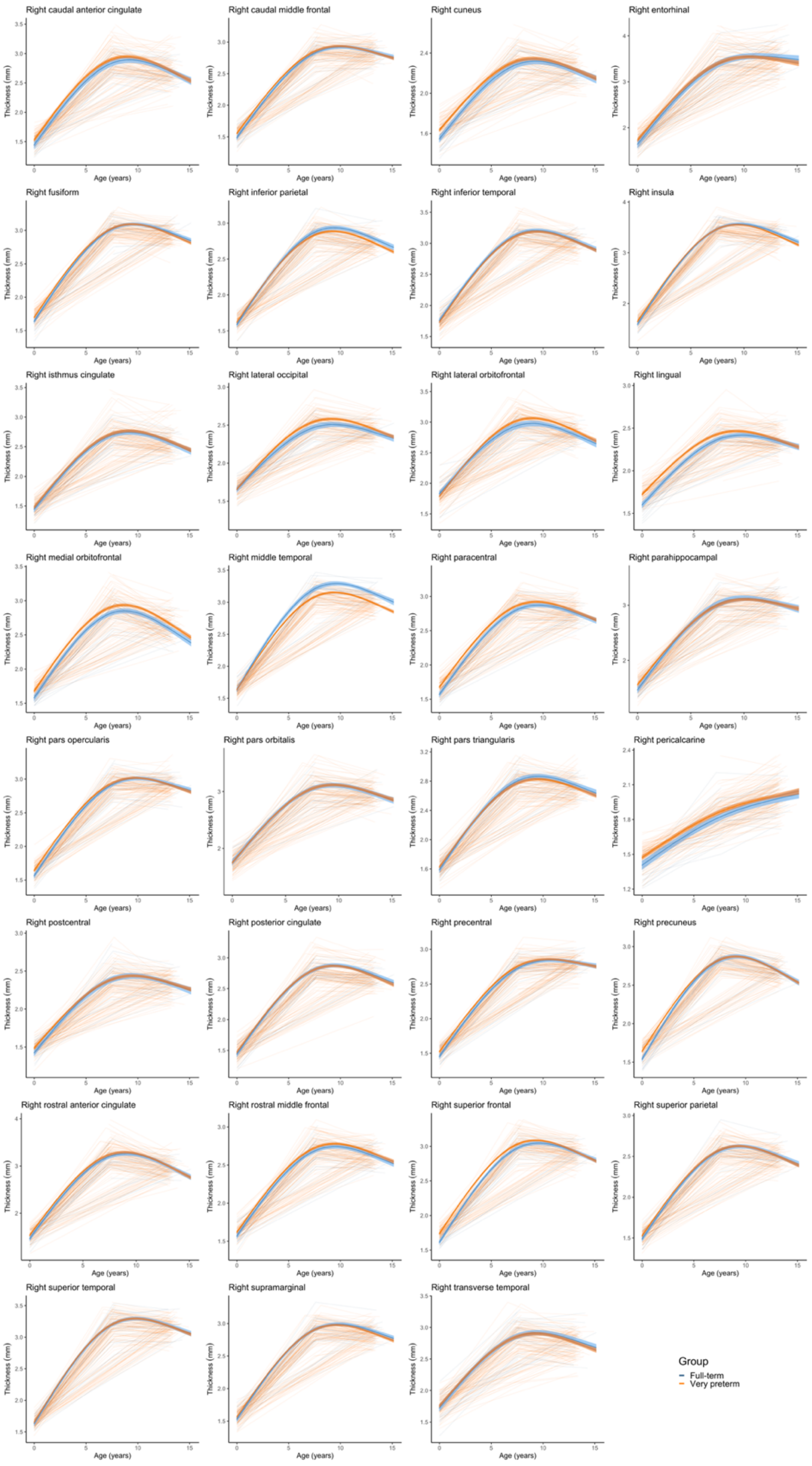

Fig. S9. Modelled trajectories by group for *thickness* of *right* hemisphere cortical regions.

This section shows group differences in cortical volume, area and thickness development, additionally adjusted for voxel size at the 0-year study point (Fig. S10).

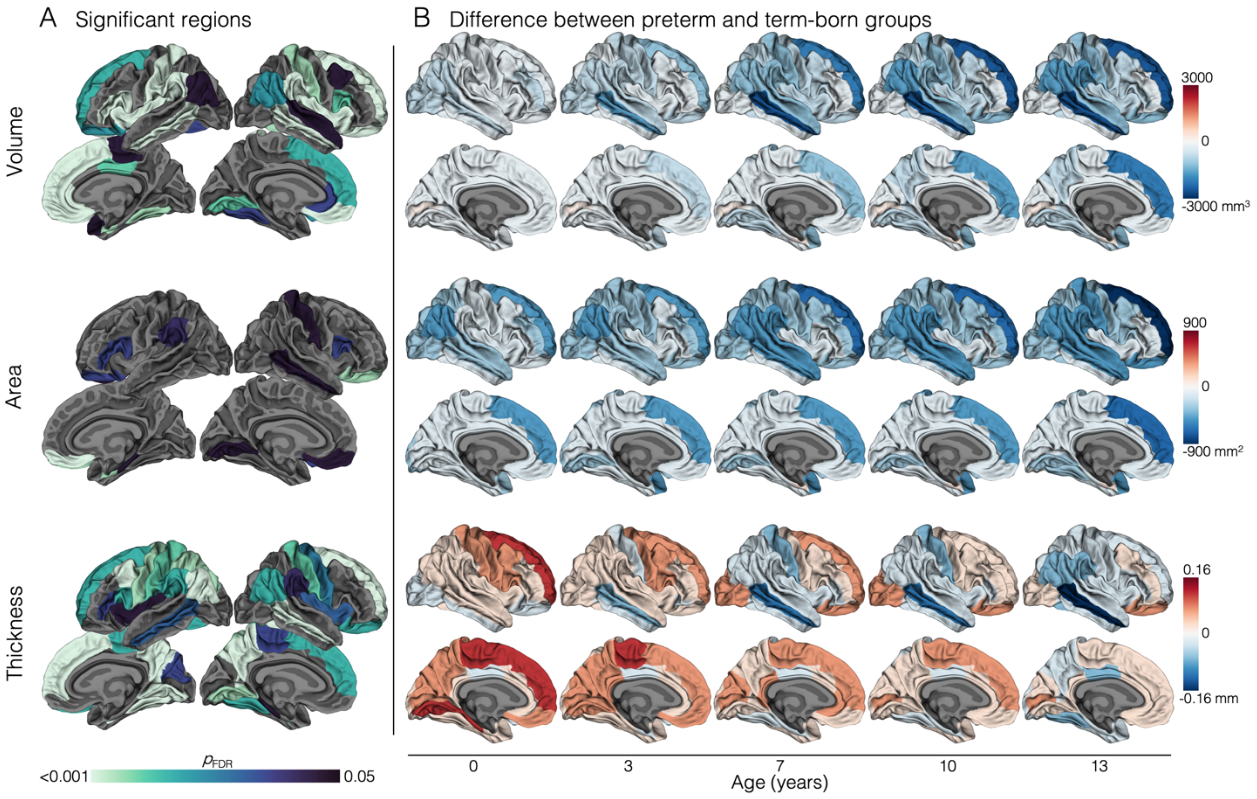

Fig. S10. Differences in cortical development between term-born and very preterm-born children, additionally adjusted for voxel size at the 0-year study point. Results were also adjusted for sex. Part A (far left) shows the cortical regions in which the longitudinal change in cortical volume, area and thickness differed between children born very preterm and children born at term (false discovery rate (FDR)-corrected $p < 0.05$). For further interpretation of these differences shown in part A, the figures in part B can be referred to. In part B, the magnitude of the difference in cortical volume, area and thickness between children born very preterm and children born at term at five ages is shown plotted on the cortical surface. The group differences at these five ages (0, 3, 7, 10, 13 years) were estimated from the statistical models (see the Methods, Statistical analysis section for more details on the modelling). The blue colour scale indicates the preterm group had lower values than the term-born group, while the red colour scale indicates the preterm group had higher values than the term-born group. The results shown in this figure are similar to the original analysis presented in the main text, which was not adjusted for voxel size at the 0-year timepoint, suggesting voxel size at the 0-year timepoint did not influence the results.

This section shows group differences in cortical volume, area and thickness development, additionally adjusted for head size (Fig. S11) and body size (Fig. S12).

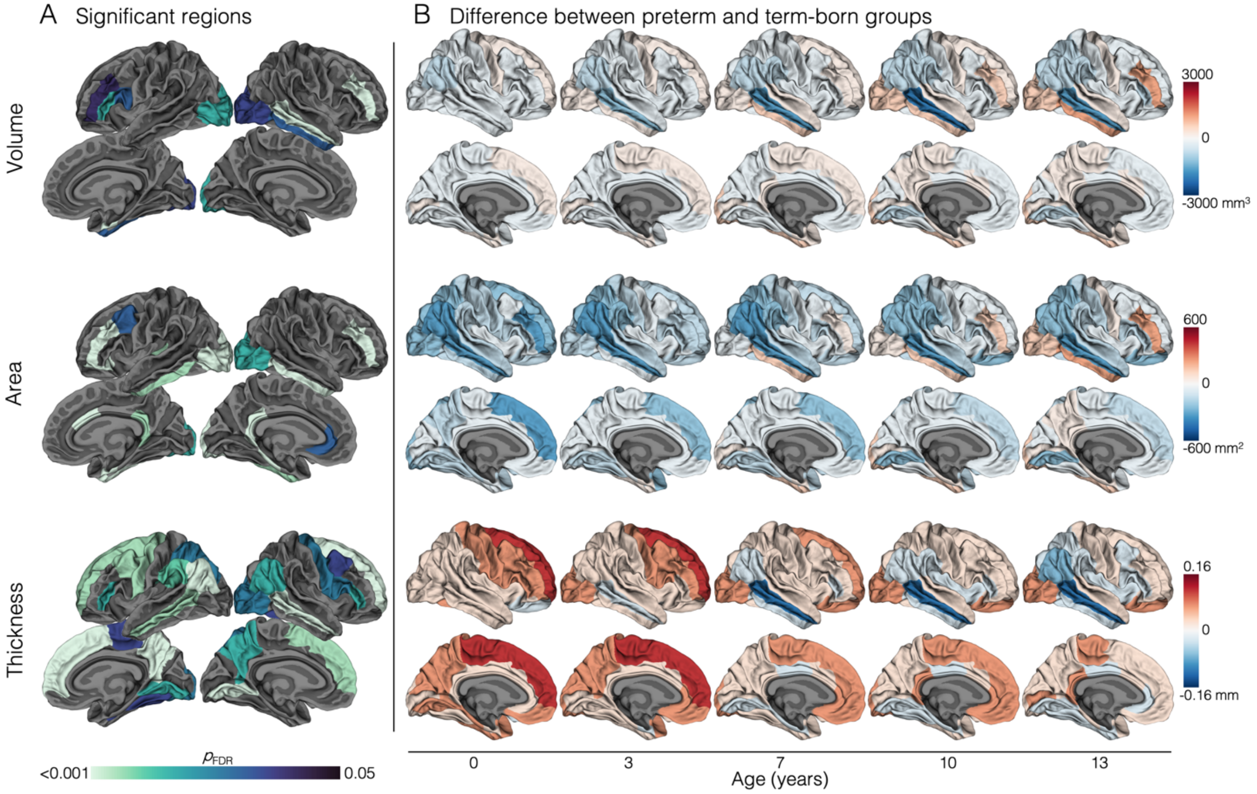

Fig. S11. Differences in cortical development between term-born and very preterm-born children, additionally adjusted for intracranial volume (ICV). Results were also adjusted for sex. Part A (far left) shows the cortical regions in which the longitudinal change in cortical volume, area and thickness differed between children born very preterm and children born at term (false discovery rate (FDR)-corrected $p < 0.05$). For further interpretation of these differences shown in part A, the figures in part B can be referred to. In part B, the magnitude of the difference in cortical volume, area and thickness between children born very preterm and children born at term at five ages is shown plotted on the cortical surface. The group differences at these five ages (0, 3, 7, 10, 13 years) were estimated from the statistical models (see the Methods, Statistical analysis section for more details on the modelling). The blue colour scale indicates the preterm group had lower values than the term-born group, while the red colour scale indicates the preterm group had higher values than the term-born group. Compared with the original analysis presented in the main text, the results in this figure varied somewhat for cortical volume and area, but not for cortical thickness. Here, the longitudinal cortical volume trajectory differed between the preterm and term-born groups for fewer regions: prominently the middle temporal region which exhibited increasingly reduced volume with age in the preterm group than the term-born group, and some frontal, occipital and inferior temporal regions which exhibited increasingly higher volume with age in the preterm group than the term-born group. For cortical area, the longitudinal trajectory differed between the preterm and term-born groups in some frontal, occipital and inferior temporal regions, where the preterm group exhibited increasingly higher area with age than the term-born group.

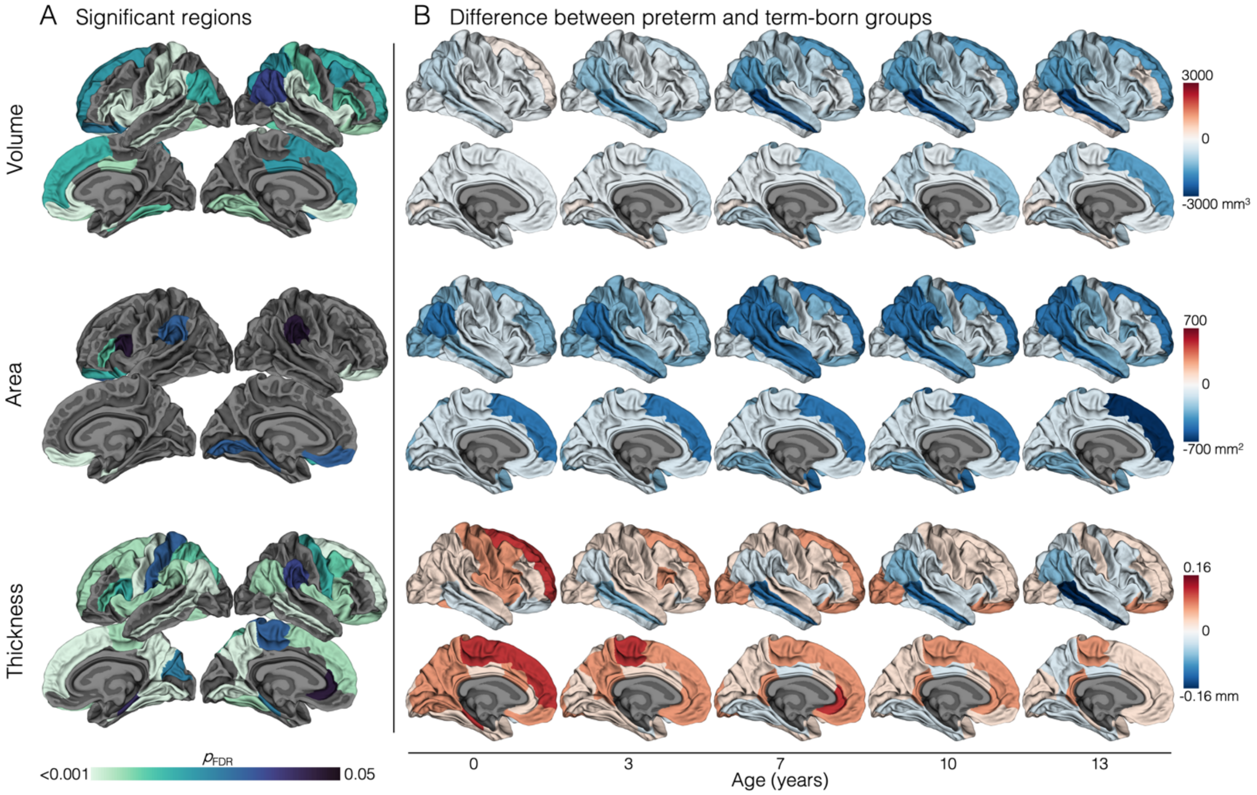

Fig. S12. Differences in cortical development between term-born and very preterm-born children, additionally adjusted for body weight. Results were also adjusted for sex. Part A (far left) shows the cortical regions in which the longitudinal change in cortical volume, area and thickness differed between children born very preterm and children born at term (false discovery rate (FDR)-corrected $p < 0.05$). For further interpretation of these differences shown in part A, the figures in part B can be referred to. In part B, the magnitude of the difference in cortical volume, area and thickness between children born very preterm and children born at term at five ages is shown plotted on the cortical surface. The group differences at these five ages (0, 3, 7, 10, 13 years) were estimated from the statistical models (see the Methods, Statistical analysis section for more details on the modelling). The blue colour scale indicates the preterm group had lower values than the term-born group, while the red colour scale indicates the preterm group had higher values than the term-born group. The results shown in this figure are similar to the original analysis presented in the main text, which was not adjusted for body weight.

Supplementary references

73. Nguyen T-N-N, Spencer-Smith M, Zannino D, *et al*. Developmental Trajectory of Language From 2 to 13 Years in Children Born Very Preterm. *Pediatrics*. 2018;141(5):e20172831. doi:10.1542/peds.2017-2831

74. Omizzolo C, Scratch SE, Stargatt R, *et al*. Neonatal brain abnormalities and memory and learning outcomes at 7 years in children born very preterm. *Memory*. 2014;22(6):605-15. doi:10.1080/09658211.2013.809765

75. Thompson DK, Matthews LG, Alexander B, *et al*. Tracking regional brain growth up to age 13 in children born term and very preterm. *Nature Communications*. Feb 4 2020;11(1):696. doi:10.1038/s41467-020-14334-9

76. Fischl B. FreeSurfer. *NeuroImage*. Aug 15 2012;62(2):774-81. doi:10.1016/j.neuroimage.2012.01.021

77. Collins SE, Thompson DK, Kelly CE, *et al*. Development of regional brain gray matter volume across the first 13 years of life is associated with childhood math computation ability for children born very preterm and full term. *Brain and Cognition*. 2022/07/01/ 2022;160:105875. doi:https://doi.org/10.1016/j.bandc.2022.105875

78. Gilchrist CP, Thompson DK, Alexander B, *et al*. Growth of prefrontal and limbic brain regions and anxiety disorders in children born very preterm. *Psychological Medicine*. 2023;53(3):759-770. doi:10.1017/S0033291721002105

79. Kelly C, Ball G, Matthews LG, *et al*. Investigating brain structural maturation in children and adolescents born very preterm using the brain age framework. *NeuroImage*. 2022/02/15/ 2022;247:118828. doi:https://doi.org/10.1016/j.neuroimage.2021.118828

80. Monson BB, Anderson PJ, Matthews LG, *et al*. Examination of the Pattern of Growth of Cerebral Tissue Volumes From Hospital Discharge to Early Childhood in Very Preterm Infants. *JAMA Pediatrics*. Aug 1 2016;170(8):772-9. doi:10.1001/jamapediatrics.2016.0781

81. Zhang Y, Inder TE, Neil JJ, *et al*. Cortical structural abnormalities in very preterm children at 7 years of age. *NeuroImage*. Apr 1 2015;109:469-79. doi:10.1016/j.neuroimage.2015.01.005

82. Bethlehem RAI, Seidlitz J, White SR, *et al*. Brain charts for the human lifespan. *Nature*. 2022/04/01 2022;604(7906):525-533. doi:10.1038/s41586-022-04554-y

83. Tamnes CK, Herting MM, Goddings A-L, *et al*. Development of the Cerebral Cortex across Adolescence: A Multisample Study of Inter-Related Longitudinal Changes in Cortical Volume, Surface Area, and Thickness. *The Journal of Neuroscience*. 2017;37(12):3402. doi:10.1523/JNEUROSCI.3302-16.2017

84. Vijayakumar N, Mills KL, Alexander-Bloch A, Tamnes CK, Whittle S. Structural brain development: A review of methodological approaches and best practices. *Developmental*

Cognitive Neuroscience. 2018/10/01/ 2018;33:129-148. doi:https://doi.org/10.1016/j.dcn.2017.11.008

85. Mills KL, Siegmund KD, Tamnes CK*, et al*. Inter-individual variability in structural brain development from late childhood to young adulthood. *NeuroImage*. Nov 15 2021;242:118450. doi:10.1016/j.neuroimage.2021.118450

86. Ball G, Seal ML. Individual variation in longitudinal postnatal development of the primate brain. *Brain Struct Funct*. Apr 2019;224(3):1185-1201. doi:10.1007/s00429-019-01829-5

87. Wood SN. Fast stable restricted maximum likelihood and marginal likelihood estimation of semiparametric generalized linear models. *Journal of the Royal Statistical Society: Series B (Statistical Methodology)*. 2011/01/01 2011;73(1):3-36. doi:https://doi.org/10.1111/j.1467-9868.2010.00749.x
